# Revisiting PFA-mediated tissue fixation chemistry: *FixEL* enables trapping of small molecules in the brain to visualize their distribution changes

**DOI:** 10.1016/j.chempr.2022.11.005

**Published:** 2023-02

**Authors:** Hiroshi Nonaka, Takeharu Mino, Seiji Sakamoto, Jae Hoon Oh, Yu Watanabe, Mamoru Ishikawa, Akihiro Tsushima, Kazuma Amaike, Shigeki Kiyonaka, Tomonori Tamura, A. Radu Aricescu, Wataru Kakegawa, Eriko Miura, Michisuke Yuzaki, Itaru Hamachi

**Affiliations:** 1 Department of Synthetic Chemistry and Biological Chemistry, Graduate School of Engineering, Kyoto University, Kyoto 615-8510, Japan; 2 ERATO (Exploratory Research for Advanced Technology, JST), Tokyo 102-0075, Japan; 3 Department of Biomolecular Engineering, Graduate School of Engineering, Nagoya University, Nagoya 464-8603, Japan; 4 Division of Structural Biology, University of Oxford, Oxford OX3 7BN, UK; 5 Neurobiology Division, MRC Laboratory of Molecular Biology, Cambridge CB2 0QH, UK; 6 Department of Neurophysiology, Keio University School of Medicine, Tokyo 160-8582, Japan

## Abstract

Various small molecules have been used as functional probes for tissue imaging in medical diagnosis and pharmaceutical drugs for disease treatment. The spatial distribution, target selectivity, and diffusion/excretion kinetics of small molecules in structurally complicated specimens are critical for function. However, robust methods for precisely evaluating these parameters in the brain have been limited. Herein, we report a new method termed “fixation-driven chemical cross-linking of exogenous ligands (*FixEL*),” which traps and images exogenously administered molecules of interest (MOIs) in complex tissues. This method relies on protein-MOI interactions and chemical cross-linking of amine-tethered MOI with paraformaldehyde used for perfusion fixation. *FixEL* is used to obtain images of the distribution of the small molecules, which addresses selective/nonselective binding to proteins, time-dependent localization changes, and diffusion/retention kinetics of MOIs such as the scaffold of PET tracer derivatives or drug-like small molecules.

## Graphical abstract

**Figure:**
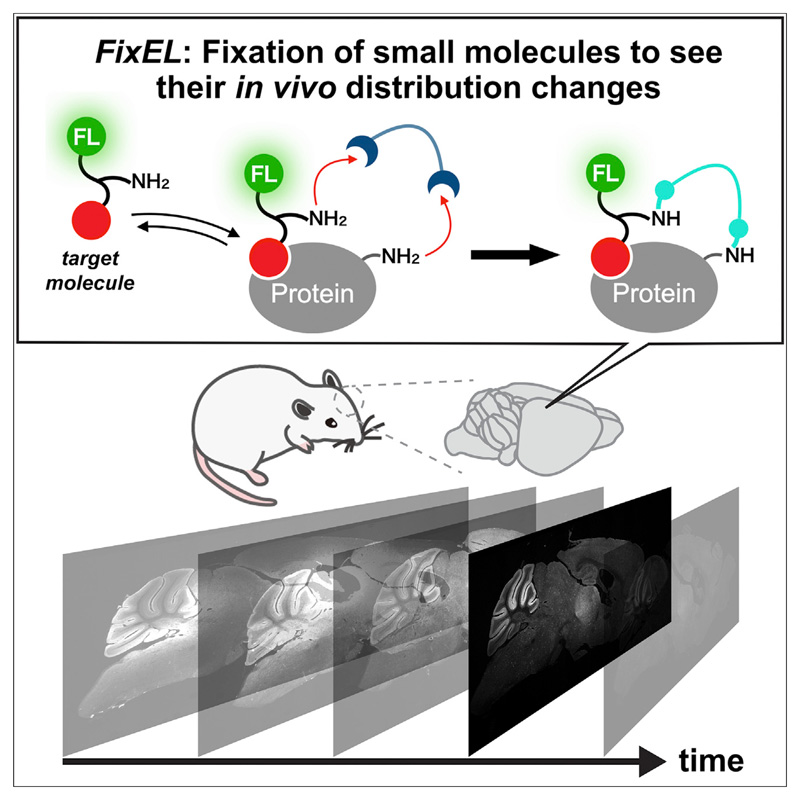
Various small molecules are used for imaging agents and medicines. Although their molecular properties such as spatial distribution *in vivo* are critical for the function, robust methods for evaluating them have been limited. Herein, we report a new method to trap and visualize exogenously administered molecules of interest (MOIs) in the brain. This method realizes to obtain images of the 3D distribution of the small molecules, which addresses selective/nonselective binding to proteins, time-dependent localization changes, and diffusion/retention kinetics of MOI.

## Introduction

**Figure 1:**
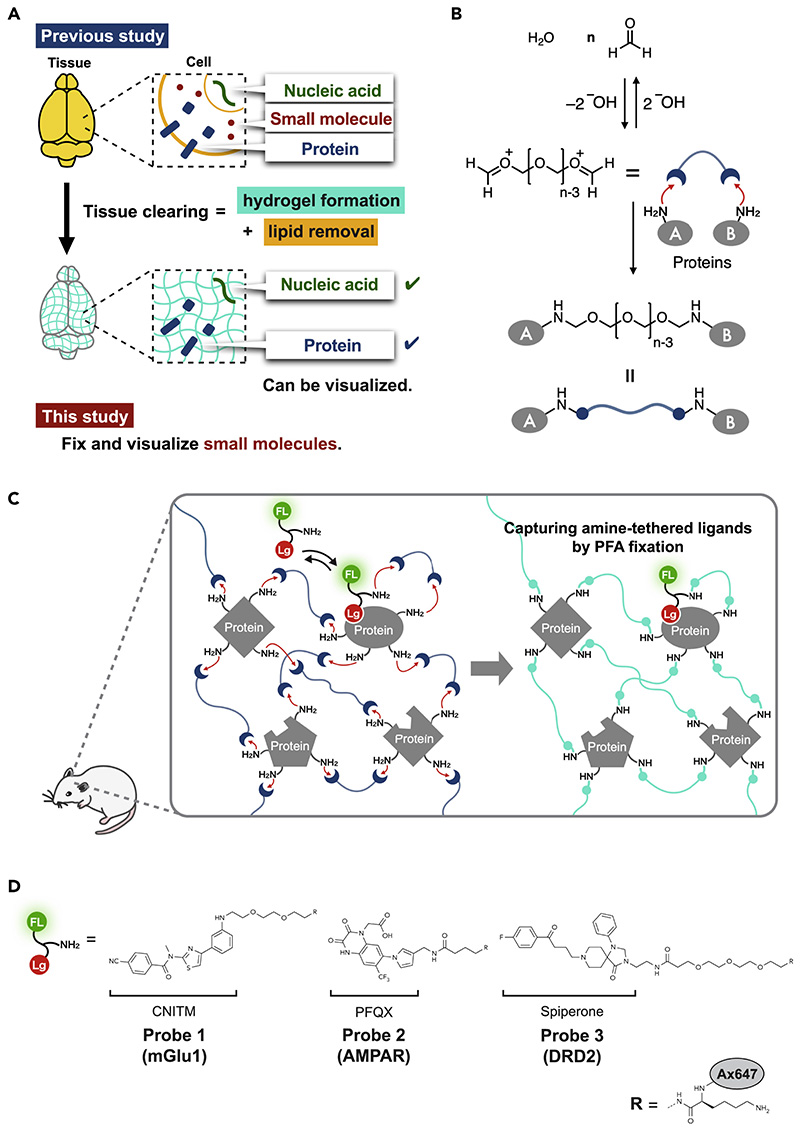
Fixation-driven chemical cross-linking of exogenous ligands (*FixEL*) in the mouse brain (A) The contribution of hydrogel-tissue chemistry (HTC) and a remaining challenge. (B) Schematic illustration of protein cross-linking by PFA. (C) Schematic illustration of the *FixEL* strategy. FL and Lg refer to fluorophore and ligand, respectively. (D) *FixEL* probes used in this study. Probe **1** (mGlu1) has a CNITM moiety, which is a ligand for mGlu1, and an amino group. Probe **2** (AMPAR) has a PFQX moiety, which is a ligand for AMPAR, and an amino group. Probe **3** (DRD2) has a spiperone moiety, which is a ligand for DRD2, and an amino group.

Tissues of living animals comprise various cells containing many of different molecules such as proteins, DNA/RNA, saccharides, and diverse small molecules. In the recent decade, the combination of hydrogel chemistry with tissues, termed hydrogel-tissue chemistry (HTC),^1–3^ has rapidly produced valuable methods capable of visualizing spatial distribution of these molecules in tissues with 3D manner, such as CLARITY,^4^ Sca*l*e,^5^ CUBIC,^6^ SeeDB,^7^ BABB,^8^ and 3DISCO.^9^ These methods physically or chemically fix and entrap protein and RNA while retaining 2D/3D arrangement of tissues within a hydrogel matrix.^10–12^ The resultant hydrogel-tissue composites are visibly clarified; as a result, the fixed proteins/DNA can be visualized in the 3D mode of the whole tissue with advanced microscopy and conventional 2D microscopy. More recently, expansion microscopy using expandable synthetic hydrogels has provided nanometer-level spatial resolution for analysis of fixed biopolymers (proteins/DNA and RNA) in a tissue composite while maintaining the 3D distribution data.^13–17^ These methods are powerful; however, they do not apply well to the 3D analysis of small molecules because most of the small molecules cannot be effectively trapped in intact tissues using the present HTC technologies (Figure 1A).

Various small molecules are used as functional probes for tissue imaging in medical diagnosis and as pharmaceutical drugs for disease treatment. These are exogenously administered to a live specimen, and they act as a reporter and/or regulator to address the normal/abnormal state of a class of cells or tissues. Spatial distribution, target selectivity, and diffusion/excretion kinetics of these molecules in structurally complicated specimens are critical for optimizing the function of small molecules. However, robust methods for precisely evaluating these parameters in tissues with high spatiotemporal resolution have been limited owing to the structural complexity, which is in sharp contrast with the tools/methods developed for cultured cell analyses. A representative imaging method is positron emission tomography (PET), which can non-invasively visualize physiological and pathological changes *in vivo* through small molecule probes labeled with radioisotopes such as ^11^C and ^18^F. Although powerful, radiolabeled molecules are difficult to handle because of the radiation exposure and short lifetime.^18,19^ PET imaging requires a special instrument for measurement and often suffers from low spatial resolution. Mass spectroscopy (MS) imaging is another useful method that detects the mass of small molecules in a tissue slice section without labeling.^20–22^ While recent progress has achieved high-resolution imaging at sub μm resolution^21^ as well as 3D analysis,^20,22^ MS imaging often requires complicated sample preparation protocols and has poor throughput in 3D analysis for thick samples.^20^ Recently reported Click-ExM^17^ and CATCH^23^ allowed researchers to visualize small molecules in cells and tissues, while their applications were limited to covalent drugs or molecules with strong affinity to biopolymers and do not cover many small molecules used in medicine and physiology, which noncovalently interact to biopolymers with moderate affinity. It remains challenging to develop a simple and general method for high-resolution 3D imaging of such small molecules in intact tissues.

Herein, we developed a new method termed “fixation-driven chemical cross-linking of exogenous ligands (*FixEL*),” which relies on interactions between proteins and small molecules and chemical cross-linking with paraformaldehyde (PFA), for imaging of exogenously administered molecules of interest (MOIs) in complex tissues such as a mouse brain. *FixEL* has been developed by revisiting the traditional PFA-based tissue fixation and combining it with a novel chemical twist, which enables to obtain snapshot images of MOI in tissues. By a combination with conventional immunostaining of sliced tissues, we are able to address the selective/nonse-lective interactions to proteins, time-dependent localization changes, and diffusion/retention kinetics of exogenously added MOIs such as the scaffold of PET tracer derivatives and drug-like small molecules. Because *FixEL* is also highly compatible with most tissue clearing technologies, the spatial distribution of MOI in the 3D mode of the whole brain was visualized with a high resolution of the synaptic puncta.

## Results

### Strategy of *FixEL* in live tissues

Perfusion fixation using PFA (formaldehyde) aqueous solution was combined with chemical cross-linking of MOI in the *FixEL* method. The formaldehyde-based tissue fixation conducted by soaking or perfusion originated in the 1890s by Ferdinand Blum et al.^24,25^ Nowadays, perfusion fixation is widely used in biology and pathology to fix tissues/cells of biological samples while maintaining the 2D/3D space.^26,27^ PFA fixation employs an aqueous formaldehyde solution consisting of polymeric formaldehyde with various degree of polymerization (Figure 1B). Aldehyde moieties of both terminals of PFA can readily react with the nucleophilic side chains (such as lysine) of many proteins in biological samples. Because most natural proteins have multiple nucleophilic amino acids and PFA is a bifunctional reactive polymer, PFA fixation produces a hydrogel-like 3D construct where PFA-modified proteins are efficiently fixed as cross-linking points (Figure 1C). However, most small molecules are not effectively trapped in this hydrogel matrix because they usually do not have such nucleophilic reactive groups and the mesh size of the resultant hydrogel is too large to entrap such small molecules. We thus decided to introduce a primary amine to MOI, to serve as a nucleophile that is reactive with PFA. Additionally, we hypothesized that attractive interactions of MOIs with proteins may assist rapid/effective cross-linking with PFA, which allows for immobilizing MOI covalently near interacting proteins during PFA-mediated fixation, as shown in Figure 1C.

A few ligand molecules that are known to selectively interact with a particular protein were tested as the MOI and modified with a reactive amino group and fluorescent reporter for imaging analysis in tissues (Figure 1D). When a modified MOI is exogenously administered to a live mouse, it reversibly interacts with a protein to form a transient complex. Upon infusion of PFA into the specimen, the amino group of MOIs are cross-linked with a nucleophilic amino acid of interacting proteins through hemiaminal bonds of PFA, resulting in covalent entrapment of the modified MOIs in the PFA-hydrogel matrix (Figure 1C). Perfusion fixation with PFA immobilizes tissues more rapidly and uniformly than with glutaraldehyde or other fixatives and allows for the capture of the living state of a specimen with minimal risk of death-induced autolysis. Subsequently, the resultant PFA-fixed tissues containing MOIs can be subjected to several analytical workflows, such as fluorescent microscopy imaging and immunostaining for studying spatial localization of MOI in sliced 2D tissues and for evaluating the 3D distribution of MOI in a mouse brain using transparent samples prepared by tissue clearing protocols. Additionally, varying the initiation time of the PFA perfusion can be used to fix distinct states of a specimen, whose tissue imaging provides valuable snapshot data, such as the time-dependent distribution of MOI, which can be correlated with its diffusion and retention rate in the whole brain.

### Proof-of-principle experiments of *FixEL* in cultured cells

**Figure 2:**
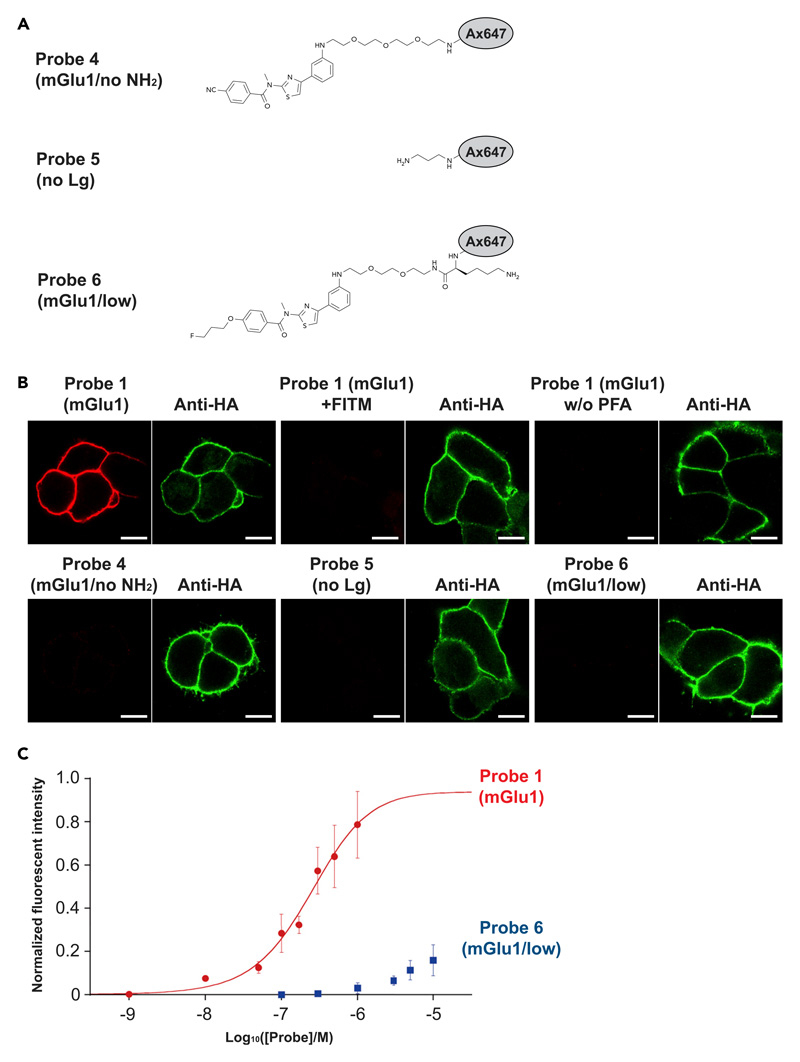
*FixEL* in cultured cells (A) Chemical structures of control molecules **4–6**. (B) Confocal imaging of HA-tag-fused mGlu1 expressed on HEK293T after *FixEL* of probe **1** (mGlu1), probe **1** (mGlu1) with 20 equiv of FITM, probe **1** (mGlu1) without PFA treating, probe **4** (mGlu1/no NH_2_), probe **5** (no Lg), or probe **6** (mGlu1/low) (Ax647, red). HA-tag-fused mGlu1 was stained with DyLight550 anti-HA tag (green). Fluorescence imaging of the cells was performed using a CLSM equipped with a 63**×** objective and a GaAsP detector (561 nm excitation for DyLight550 and 633 nm excitation for Ax647). Scale bars, 5 μm. (C) Fluorescence change of the plasma membrane on mGlu1-expressed HEK293T cells depending on the concentration of probe **1** (mGlu1) (red circle) or probe **6** (mGlu1/low) (blue square). n = 8 cells in a single dish. Data are presented as mean ± SEM.

As a proof-of-principle study, the process was initiated with 4-cyano-*N*-[4-(6-(isopropylamino)-pyrimidin-4-yl)-1,3-thiazol-2-yl]-*N*-methylbenzamide (CNITM). CNITM is a derivative of a PET probe for metabotropic glutamate receptor type 1 (mGlu1),^28^ a type-C G protein-coupled receptor (GPCR) expressed in the cerebellum and thalamus regions, whose expression level may be related to Parkinson’s disease.^29,30^ CNITM bearing the affinity with mGlu1 (*K*_i_ = 27 nM), was modified with a primary amine and a fluorescent Alexa Fluor 647 (Ax647) dye to produce the CNITM probe **1** (mGlu1) (Figure 1D). The accessible site for such modification was judged from the co-crystal structure of mGlu1 and 4-Fluoro-N-[4-[6-(isopropylamino)pyrimidin-4-yl]-1,3-thiazol-2-yl]-N-methylbenzamide (FITM), a ligand similar to CNITM.^31^ Three control molecules shown in Figure 2A were also prepared.

*FixEL* experiments were first conducted using HEK 293T cells expressing mGlu1 (Figure 2B). After incubation of mGlu1-expressing cells with probe **1** (mGlu1) for 5 min, the cells were treated with PFA (30 min), followed by washing with DMEM (37°C, overnight). The fluorescence of Ax647 was observed from the plasma membrane of mGlu1-expressing cells using confocal laser scanning microscopy (CLSM). Such fluorescence was not observed with probe **4** (mGlu1/no NH_2_) that lacks the amine moiety, clearly indicating the reactive amine is essential for this cross-linking. Under the conditions where FITM bearing the stronger affinity than CNITM was co-incubated with probe **1** (mGlu1), the fluorescence was nearly diminished. Negligible fluorescence was detected in the case of probe **5** (no Lg) with an amine and Ax647 and without appropriate ligand. Therefore, the CNITM-mGlu1 interaction is also essential for the cross-linked fixation of probe **1** (mGlu1). There was no fluorescence at the plasma membrane in the absence of PFA (Figure 2B). Moreover, probe **6** (mGlu1/low) with a lower affinity ligand (*K*_i_ > 5 μM)^32^ also showed negligible fluorescence on the cell membrane. Therefore, the probe **1** (mGlu1) fixation was carried out by the amine-originated cross-linking of PFA with the essential assistance of sufficient protein-ligand interaction. In the titration experiment with varied concentration of probe **1** (mGlu1), the fluorescence intensity from the plasma membrane showed a typical saturation behavior with 243 ± 77 nM of *K*_d_, which is slightly larger than that of CNITM,^28^ confirming the binding of probe **1** (mGlu1) with mGlu1 is a key control factor in this cross-linked fixation (Figure 2C). On the other hand, the fluorescence intensity from the probe **6** bearing a low-affinity ligand (mGlu1/low) did not show saturation behavior up to 10 μM, indicating that *FixEL* can reflect the affinity of MOI to a binding protein.

Based on our obtained results that a reactive amine is essential for the trapping of small molecules by *FixEL*, we next sought to construct a new type of fixation-based sensor detecting the environmental change in the proximity to a target protein. We newly designed a *FixEL* reagent where the amine group was masked with an appropriate protective group cleavable in response to a stimulus. It was expected that a given specific stimulation cleaved off the protective group so that a reactive amine is exposed, allowing the cross-linking of the reagent with a protein-of-interest by PFA (Figure S1A). To demonstrate this idea, we synthesized a new mGlu1 ligand-based *FixEL* reagent bearing an H_2_O_2_-responsive phenylboronic acid as a protective group of the amine functionality (Figure S1B). As shown in Figures S1C-S1E, this *FixEL* probe is fixed on mGlu1-expressed cells only in the presence of H_2_O_2_ in a dose dependent manner. This result indicates the *FixEL* strategy may be extended to new class of fixation-driven chemical sensing.

### *FixEL* of the small molecule probe for mGlu1 in a mouse cerebellum

**Figure 3:**
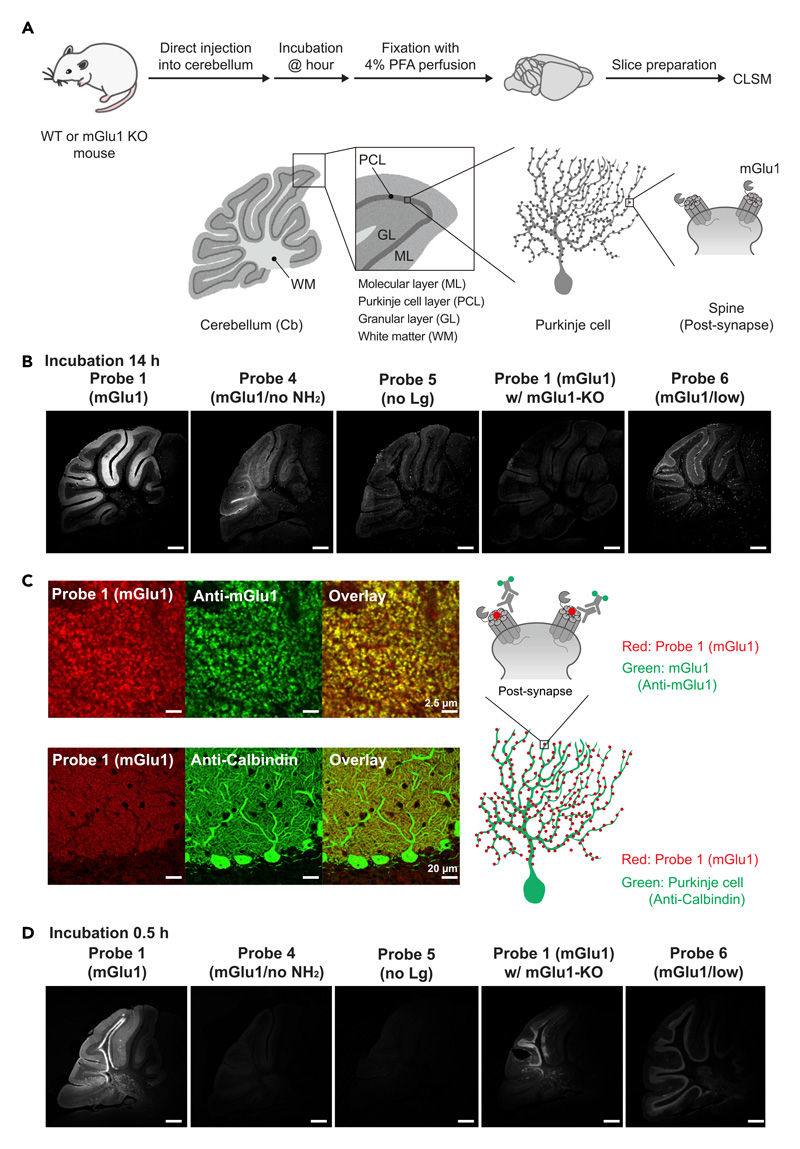
*FixEL* of a small molecule ligand for mGlu1 in the mouse cerebellum (A) Schematic illustration of experimental procedures. PBS(–) containing probe **1** (mGlu1) (4.5 μL of 10 μM probe which is assumed to be ca. 0.7 μM (C_cere_) on the basis of the cerebellum volume^33^) was injected into mouse cerebellum. After 0.5 or 14 h of incubation, the mouse was transcardially perfused with 4% PFA. The cerebellum was isolated and sectioned by cryostat (50-μm thick). (B) Fluorescence imaging of cerebellum slices with *FixEL* of probe **1** (mGlu1) after 14 h incubation. Imaging was performed using a CLSM equipped with a 10**×** objective and a GaAsP detector (633 nm excitation for Ax647). From left to right images, probes **1** (mGlu1), **4** (mGlu1/no NH_2_), **5** (no Lg), and probe **1** (mGlu1) in mGlu1 KO mouse, and probe **6** (mGlu1/low) were used. Scale bars, 500 μm. (C) Co-immunostaining of the cerebellum slice with *FixEL* of probe **1** (mGlu1) after 12 h incubation. Probe **1** (mGlu1) is shown in red. Anti-mGlu1 and anti-calbindin are shown in green. The slices after *FixEL* of probe **1** (mGlu1) were permeabilized and immunostained with primary antibody anti-mGlu1 or anti-calbindin and secondary antibody (Alexa Fluor 488 [Ax488]) in 0.1% Triton X-100/PBS(–). Fluorescence images in top and bottom were acquired by using a CLSM equipped with a 100**×** objective, a GaAsP detector (488 nm excitation for Ax488 and 633 nm excitation for Ax647), and lightning deconvolution. Fluorescence image in middle was acquired by using a CLSM equipped with a 63**×** objective and a GaAsP detector (488 nm excitation for Ax488 and 633 nm excitation for Ax647). (D) Fluorescence imaging of cerebellum slices with *FixEL* of probe **1** (mGlu1) after 0.5 h incubation. Imaging was performed using a CLSM equipped with a 5**×** objective. Scale bars, 500 μm. See also Figure S3.

*FixEL* was used in a mouse cerebellum whose molecular layer highly expressed endogenous mGlu1, to examine the spatial distribution of probe **1** (mGlu1). Probe **1** (mGlu1) in an aqueous solution was injected directly into the cerebellum of 5-week-old live C57BL/6N mice under anesthesia; thereafter, we confirmed that the mice were alive and freely moved. Perfusion fixation by PFA was performed 14 h after the probe injection, and the fixed cerebellum was cut into 50-μm thick sections by cryostat (Figure 3A). CLSM observation of the sections showed that Ax647-derived fluorescence was observed selectively in the molecular layer region, which was in good agreement with the expression area of mGlu1 (Figure 3B).^34^ We confirmed that this fluorescence signal is derived from probe **1** (mGlu1) itself but not from its decomposed products, by the HPLC analysis of the mouse cerebellum homogenate before PFA fixation (Figure S2). Clear and selective fluorescence was not observed in the cases of probes **4** (mGlu1/no NH_2_) or **5** (no Lg) that lack either the amine group or ligand part, respectively. Similarly, low-affinity probe **6** (mGlu1/low) also exhibited much weaker signals than probe **1** (mGlu1). Additionally, in the experiment using mGlu1 KO mice, the fluorescence signal was strongly diminished, which suggests that probe **1** (mGlu1) was fixed proximal to mGlu1, and both the amine tethering and ligand-protein interaction are essential for the effective entrapment of probe **1** (mGlu1).

High-resolution CLSM imaging confirmed that the fluorescence in the molecular layer comprised an assembly of many bright puncta with μm size, which is consistent with the perisynaptic localization of mGlu1 (Figure 3C). The dendritic spines derived from Purkinje cells were stained with anti-Calbindin, a Purkinje cell marker. Immunostaining of mGlu1 showed that many of the bright spots of Ax647 merged well with the fluorescence from anti-mGlu1. All of these results strongly demonstrate that the location of the CNITM probe **1** (mGlu1) was visualized by *FixEL*, and probe **1** (mGlu1) was retained proximal to mGlu1 in the molecular layer of the cerebellum through CNITM-mGlu1 selective interactions for at least 14 h in the live mouse.

Because nonspecific interactions of small molecules in tissues impact their diffusion, retention, and functions in many cases, it is crucial to evaluate such nonspecific interactions with off-target molecules, as well as the specific one. We here observed a weak fluorescence signal even in the brain slices prepared from mGlu1 KO mouse, which should be due to the nonspecific binding of probe **1** (mGlu1) (Figure 3B). This result indicates that *FixEL* allows the visualization of nonspecific interactions of small molecules in the brain tissue. On the basis of this feature, we attempted to observe the brain slice fixed immediately after probe administration. The short incubation time (0.5 h) after the probe **1** (mGlu1) injection produced a rather non-characteristic image near the injection site including specific and nonspecific binding of the probe, in which the fluorescent areas were broadly spread in the granular layer and white matter, other than the molecular layer (Figure 3D). This image may be explained by that an excess amount of probe **1** (mGlu1) remained in the injection area in an early time after the injection, and many of probe **1** (mGlu1) can be fixed by PFA perfusion through nonspecific interactions with proteins other than mGlu1, as well as specific binding to mGlu1. Given that such images were not obtained for the control probes **4** (mGlu1/no NH_2_) or **5** (no Lg), the nonspecific interactions reflect the distinct physicochemical properties of the ligand including the hydrophobicity and presence/absence of the tethered amine. Such non-characteristic signals were reduced after 14 h incubation, and selective fluorescence of the molecular layer appeared. In the case of probe **6** (mGlu1/low), a short incubation time (0.5 h) produced weak fluorescence in the granular layer and Purkinje cell bodies as with the KO sample, indicating the poor targetability of this weaker affinity ligand. These results demonstrate that *FixEL* can be useful for evaluating both specific and nonspecific interactions of small molecules in tissues by fluorescence imaging.

The diffusion behavior of probe **1** (mGlu1) was visualized using the distance-dependent imaging data (Figure S3). The perfusion fixation was performed 12 h after the injection, followed by slices 0.6, 1.2, and 1.8 mm from the right side of the injection point, the cerebellar vermis, 0 mm. Selective fluorescence of the molecular layer was observed in all slices, while the signal intensity gradually decreased depending on the distance from the injection point, which implies that probe **1** (mGlu1) penetrated, diffused, and retained along the direction away from the injection site and a concentration of probe **1** (mGlu1) reduced around the edges of the cerebellum.

### *FixEL* to visualize MOI of mGlu1 in the whole brain

**Figure 4:**
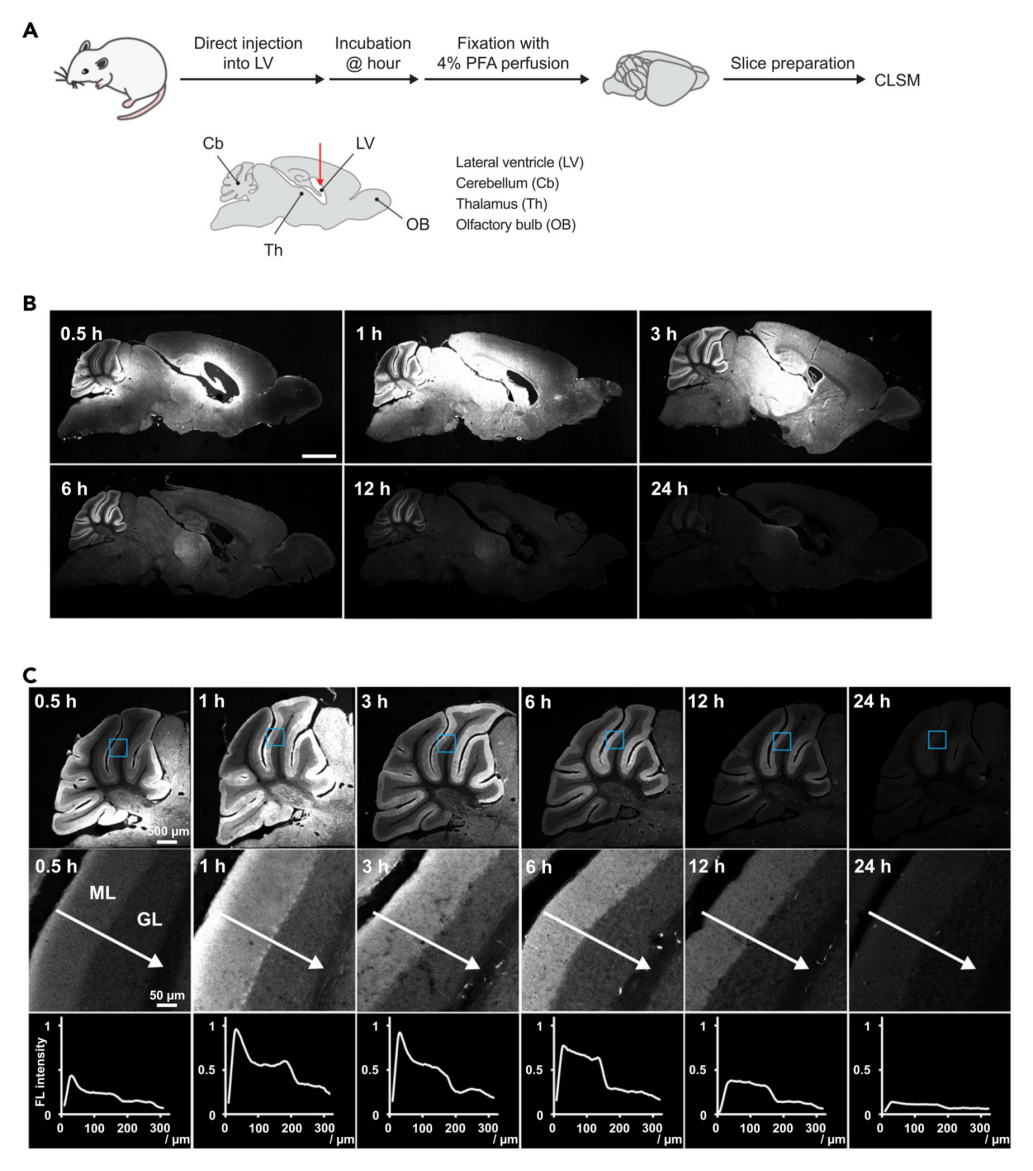
Visualization of the distribution changes of probe **1** (mGlu1) after *FixEL* at different incubation times (A) Schematic illustration of experimental procedures. Fluorescence imaging of whole-brain sagittal slices after *FixEL* with probe **1** (mGlu1). PBS(–) containing probe **1** (mGlu1) (4.5 μL of 100 μM probe which is assumed to be ca. 1 μM [C_brain_] based on the brain volume^33^) was injected into the mouse lateral ventricle. After 0.5, 1, 3, 6, 12, or 24 h of incubation, the mouse was transcardially perfused with 4% PFA. (B) Fluorescence imaging of slices (50-μm thick) was performed using a CLSM equipped with a 5**×** objective and a GaAsP detector (633 nm excitation for Ax647). Scale bars, 2 mm. (C) Line plot analysis of distribution changes of probe **1** (mGlu1) in the cerebellar lobule V after injection into the lateral ventricle. Fluorescence imaging of the cerebellum region was performed using a CLSM equipped with a 10**×** objective. The light blue square region was magnified. The line plot analysis was performed along a white arrow. ML, molecular layer; GL, granular layer.

The direct injection of probe **1** (mGlu1) from the lateral ventricle (LV) allowed for analyzing its spatial distribution in the whole brain (Figures 4A and 4B). Various molecules, such as peptides, proteins, and DNA/RNA, injected into LV are diluted by the cerebrospinal fluid and diffuse with its flow. After injection of probe **1** (mGlu1) into the LV, perfusion fixation with 4% PFA was performed with incubation times of 0.5, 1, 3, 6, 12, and 24 h. Sections 600 μm from the brain midline were prepared and observed using CLSM (Figure 4B). mGlu1 is expressed in the thalamus and olfactory bulb, as well as the cerebellum of the whole brain. For the slice fixed 0.5 h after injection, strong fluorescence was observed near the LV region and at the outer edge of the cerebellum where the cerebrospinal fluid is in contact. The slice after 1 h incubation showed widely distributed fluorescence from the LV region to the thalamus and mid-brain areas, suggesting that the probe penetrated the brain parenchyma and was trapped there through cross-linking with nonspecific interactions caused by the high concentration of probe **1** (mGlu1), whereas the fluorescence derived from the selective interaction to mGlu1 became clearer in the cerebellar molecular layer. In the slice after 3 h of incubation, the overall fluorescence intensity was less than that of shorter incubations, probably because of the washing-out effect with cerebrospinal fluid. Additionally, the selective staining of the molecular layer in the cerebellar region was more clearly visualized while the fluorescence due to nonspecific interactions still remained in the mid-brain areas. The fluorescence reflecting the mGlu1 expression areas in the molecular layer of the cerebellum and thalamus gradually faded 6–24 h after injection, whereas the fluorescence in other areas nearly disappeared at 6 h. This suggests that the probe fixed due to nonspecific interactions was more rapidly excreted with the flow of cerebrospinal fluid. The snapshot images with high resolution allowed us to chase the diffusion of probe **1** (mGlu1) more closely (Figure 4C). At the edge of the cerebellar molecular layer that is the contact surface with the cerebrospinal fluid, the fluorescence strongly appeared 0.5 h after injection and intensified over 3 h. The fluorescence at the interface between the molecular layer and granular layer was weak relative to that of the contact area, resulting in a fluorescence gradient from the contact area to the inner interface. The gradient gradually became gentle and flat over 12 h. This implied that probe **1** (mGlu1) permeated from the contact area, diffused to the molecular layer, and distributed homogeneously in the molecular layer; however, probe **1** (mGlu1) was not trapped in the granular layer; thereafter, the probe was slowly discharged. These results suggest that *FixEL* is a unique technique capable of visualizing the diffusion snapshots of MOI, as well as the spatial distribution of MOI in the whole brain with high resolution.

### *FixEL*-based imaging of various MOI probes in the whole brain

**Figure 5:**
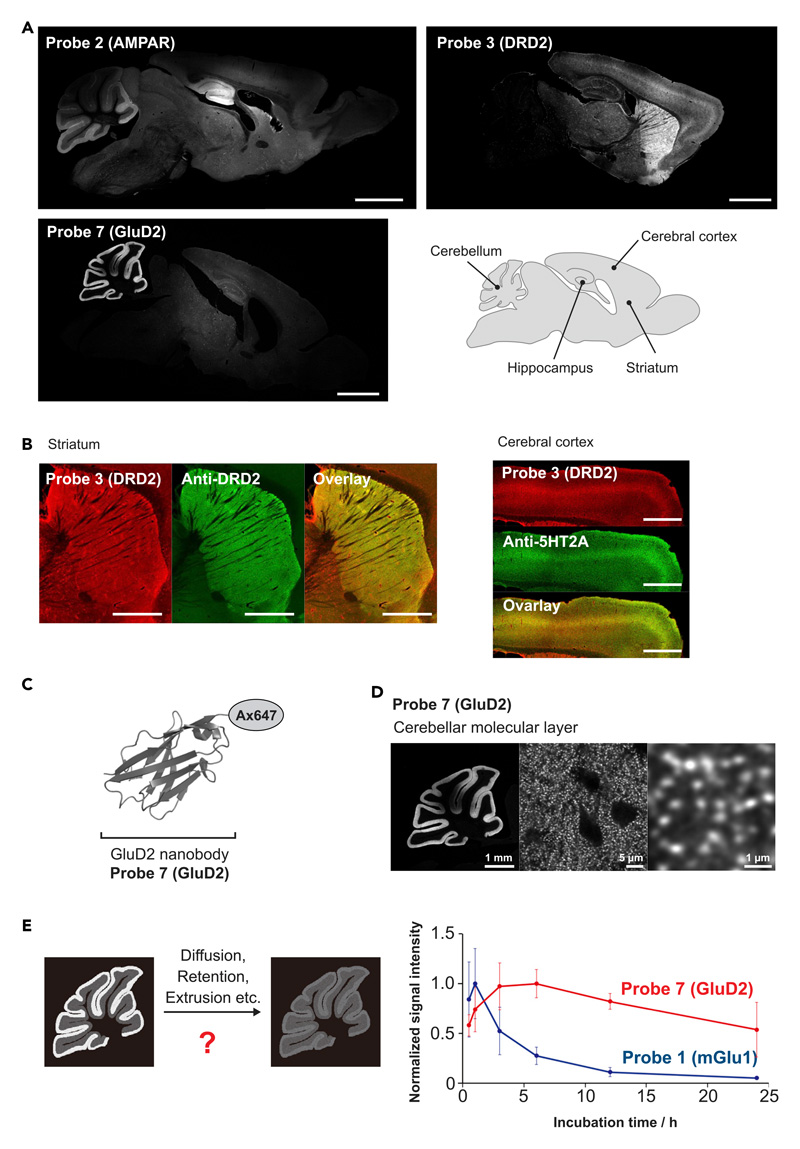
Distribution and localization analysis of *FixEL* probes **2** (AMPAR), **3** (RDR2), and **7** (GluD2) in the whole brain (A) Fluorescence imaging of whole-brain sagittal slices after *FixEL*. PBS(–) containing a probe (4.5 μL) was injected into the mouse lateral ventricle. Conditions: 40-μM probe **2** (AMPAR) (C_brain_ is ca. 0.4 μM), incubation time 3 h. 25-μM probe **3** (DRD2) (C_brain_ is ca. 0.25 μM), incubation time 16 h. 40 μM probe **7** (GluD2) (C_brain_ is ca. 0.4 μM), incubation time 24 h. Fluorescence imaging of slices (50-μm thick) was performed using a CLSM equipped with a 10**×** or 5**×** objective and a GaAsP detector (633 nm excitation for Ax647). Scale bars, 2 mm. (B) Co-immunostaining of the slices after *FixEL* with probe **3** (DRD2). The slices were fixed, permeabilized, and immunostained using anti-DRD2 or anti-5HT2A (green, Ax488-conjugated secondary antibody). Scale bars, 1 mm. (C) An Ax647-conjugated GluD2 nanobody, probe **7** (GluD2). The model illustration of nanobody was from PDB 4zg1. (D) Higher magnification image of the sample in (A) stained with probe **7** (GluD2). Fluorescence imaging was performed using a CLSM equipped with a 10**×** objective (left panel) and a 100**×** objective with lightning deconvolution (middle and right panel). (E) Comparison of the time-dependent decrease in fluorescence of cerebellar regions between slice samples with *FixEL* using probe **1** (mGlu1) (blue circle) and probe **7** (GluD2) (red circle). The average values of the signal intensities of the four ROIs on the cerebellar molecular were normalized to their maximum value. Data are presented as mean ± SEM. See also Figures S4 and S7.

*FixEL* was applied to other small molecular ligands, such as 6-pyrrolyl-7-trifluoromethyl-quinoxaline-2,3-dione (PFQX) and spiperone to demonstrate the robustness of the method. PFQX is a selective antagonist of the α-amino-3-hydroxy-5-methyl-4-isoxazolepropionic acid (AMPA) receptor (AMPAR), an ionotropic glutamate receptor.^35–37^ According to the design guideline of probe **1** (mGlu1), a PFQX-tethered probe **2** (AMPAR) was chemically synthesized (Figure 1D). 3 h after direct injection of probe **2** (AMPAR) into LV, perfusion fixation was conducted, and the slice samples were prepared. Fluorescence signals were mainly observed in the hippocampus, cerebellum, and cortex where AMPARs are naturally expressed (Figure 5A). We confirmed that the fluorescence signal of probe **2** (AMPAR) merged with the immunostaining signal of anti-GluA2 (Figure S4A). Spiperone, which has been used as a psychiatric drug, is an antagonist of the D2 dopamine receptor (DRD2) and ^11^C-*N*-methylspiperone is used as its PET tracer.^38–40^ We similarly synthesized a spiperone-tethered probe **3** (DRD2) (Figure 1D). The fluorescence signals of the slice prepared by the *FixEL* protocol (16 h after injection) were clearly observed in the striatum where endogenous D2 receptors are expressed (Figure S4B). We also detected fluorescence in the middle of the cerebral cortex. Because spiperone is also known to bind to the 5HT2A serotonin receptor expressed in the cerebral cortex, this fluorescence might be derived from the spiperone-5HT2A interaction (*K*_i_ for DRD2 = 0.06 nM, *K*_i_ for 5HT2A ≈ 1–2 nM).^41–43^ The fluorescence signal of probe **3** (DRD2) merging with the immunostaining signal was confirmed using anti-DRD2 in striatum region and 5HT2A in cerebral cortex region (Figure 5B), demonstrating *FixEL* provides the ability to image the distribution of MOI relying on the interaction not only with the target protein but also the off-target protein in the brain.

Dual-color imaging is powerful because it can directly compare the distribution of different molecules within the same individual. *FixEL* probes of mGlu1 ligand bearing Ax555 and DRD2 ligand bearing Ax647 (probe **3** (DRD2)) were co-injected into the LV of a single mouse. As shown in Figure S5, the clearly distinct fluorescence signals between Ax555 and Ax647 were observed in the cerebellum and striatum regions, respectively, which is in good agreement with the imaging data of the single *FixEL* probe. The result demonstrates that *FixEL* allows multi-component analysis in a single specimen.

Since it was demonstrated that *FixEL* enables an evaluation of the time-dependent distribution changes, we next attempted to compare the diffusion properties of two MOIs having largely different characteristics in the molecular weight (MW) and affinity. In addition to a small molecule ligand CNITM, we employed nanobody, a downsized antibody. Similar to mGlu1, GluD2, a glutamate receptor, is endogenously located at the post-synapse of the cerebellar molecular layer,^44^ and we used GluD2 nanobody shown in Figure 5C.^45,46^ GluD2 nanobody was conjugated with Ax647, yielding probe **7** (GluD2) of 15 kDa of MW and 34.7 nM of *K*_d_, which are 10-fold higher and 6-fold smaller than those of CNITM-*FixEL* probe, respectively (Figure S6). Probe **7** (GluD2) was injected into the LV of 5-week-old C57BL/6N mice followed by perfusion fixation (24 h), and the slice samples were prepared, according to the *FixEL* protocol. Strong fluorescence signal was observed predominantly in the cerebellar molecular layer of the slices (Figure 5A) that merged well with anti-GluD2 (Figure S4C). The signal comprised many small bright puncta smaller than 1 μm (Figures 5D and S4D). Similar to CNITM probe **1** (mGlu1), it was possible to capture time-dependent changes in the distribution of GluD2 nanobody **7** (GluD2) by varying the perfusion fixation time after injection into the LV (Figure S7). The time profile of the fluorescence intensity of CNITM probe **1** (mGlu1) in the cerebellar molecular layer showed a biphasic behavior including the fluorescence increase in the initial 1 h and subsequent decrease, indicating that probe **1** (mGlu1) penetrated/condensed up to 1 h and then was slowly excreted with a half-life of approximately 6 h (Figure 5E). On the other hand, Ax647-GluD2 nanobody showed a rather slow increase within 3–6 h and decrease with a halftime of longer than 24 h. This difference in CNITM and GluD2 nanobody retention time in the cerebellar molecular layer may be attributed to the dissociation rate of the corresponding ligand-protein interaction (i.e., CNITM-mGlu1 versus nanobody-GluD2). We separately confirmed the much more rapid dissociation of CNITM from mGlu1, compared with that of Ax647-GluD2 Nb from GluD2 using the live cell experiments (Figure S8). The results indicate that the *FixEL* method has the potential to directly evaluate parameters related to the diffusion of MOIs in the whole brain of live mice.

### Transparent tissues prepared by combining *FixEL* and 3DISCO

**Figure 6:**
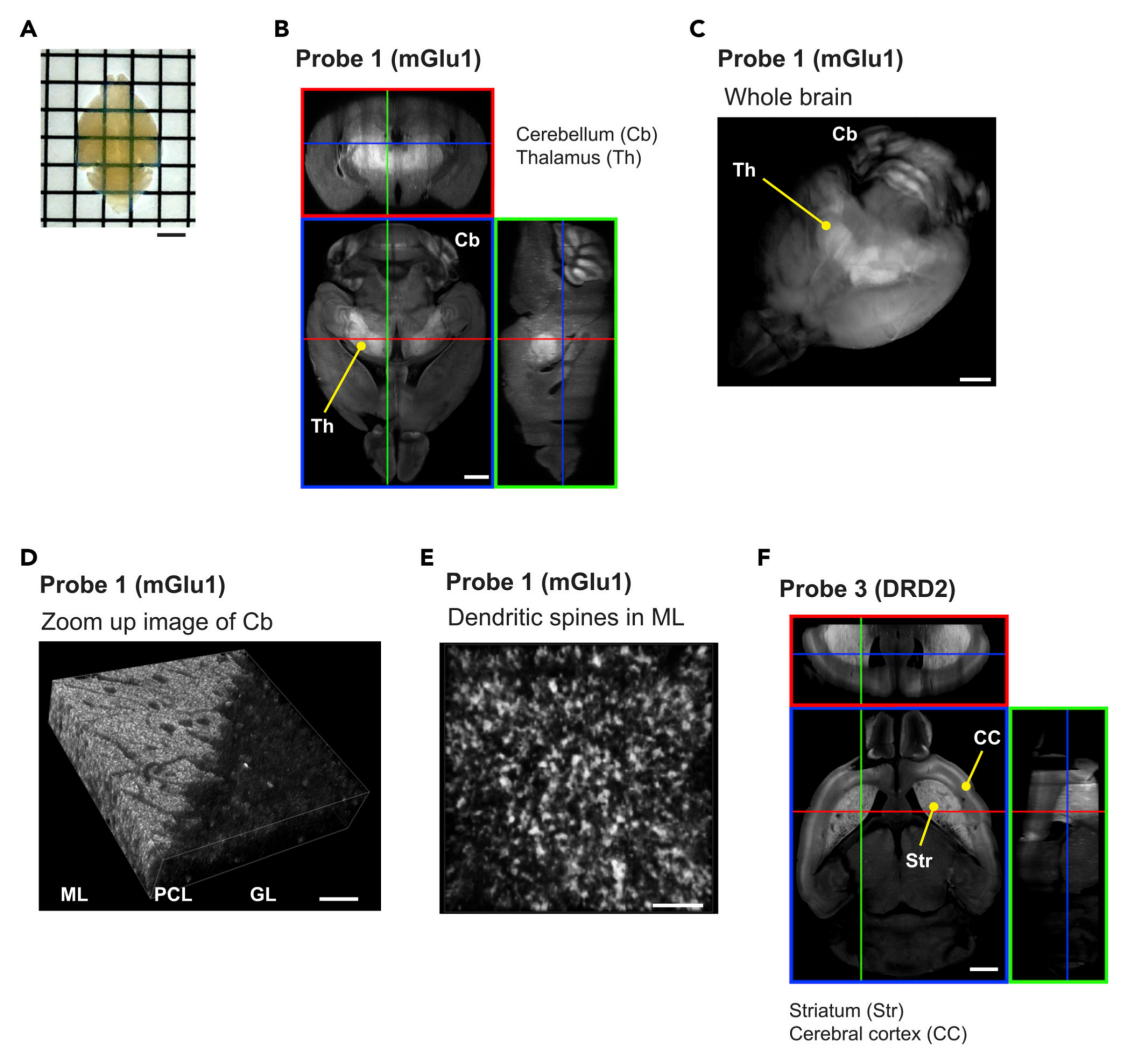
3D fluorescence imaging of *FixEL* samples with 3DISCO (A) Appearance of a brain sample after *FixEL* with probe **1** (mGlu1) and 3DISCO. Scale bars, 2.5 mm. (B) z stacking fluorescence imaging of the (A) sample. PBS(–) containing probe **1** (mGlu1) (4.5 μL, 100 μM: C_brain_ is ca. 1 μM) was injected into mouse lateral ventricle. After 6 h of incubation, the mouse was transcardially perfused with 4% PFA. After 3DISCO treatment, z stacking fluorescence imaging of the whole brain was performed using a CLSM equipped with a 5**×** objective and a GaAsP detector (633 nm excitation for Ax647). Scale bars, 1 mm. (C) 3D rendering of the whole brain in (B). Scale bars, 1 mm. (D) z stacking fluorescence imaging of cerebellum region in (C) with a 40**×** objective. Scale bars, 20 μm. (E) Enlarged 3D rendering image of the cerebellar molecular layer (ML) in (D). Scale bars, 5 μm. (F) z stacking fluorescence imaging of the whole brain after *FixEL* with probe **3** (DRD2). PBS(–) containing 25 μM of probe **3** (DRD2) (4.5 μL) was injected into mouse bilateral ventricle respectively. After *FixEL* (20 h incubation), the lower 2 mm of the brain was cutoff and then the upper side of the brain was treated with 3DISCO. z stacking fluorescence imaging was performed using a CLSM equipped with a 5**×** objective and a GaAsP detector (633 nm excitation for Ax647). Scale bars, 1 mm. See also Figures S9-S11.

The fixed tissue prepared by *FixEL* with probe **1** (mGlu1) was compatible with the tissue clearing protocol of 3DISCO.^9^ The cerebellum tissue was made transparent by 3DISCO, and the resultant tissue sample was subjected to 3D CLSM imaging, showing that probe **1** (mGlu1) was selectively and three-dimensionally distributed in multiple molecular layers throughout the whole cerebellum (Figure S9). A whole brain sample prepared by the LV injection of probe **1** (mGlu1) was also clarified and 3D CLSM imaging was performed (Figures 6A-6E). Clear fluorescence was observed in the thalamus and cerebellar molecular layer where mGlu1 was localized (Figures 6B and 6C). When the cerebellar molecular layer of this transparent sample was observed with a higher magnification lens of CLSM, the molecular layer was observed to be densely packed with bright puncta smaller than 1 μm, likely derived from dendritic spines (Figure 6E). To demonstrate the versatility of 3D distribution analysis by combining *FixEL* and 3DISCO, we also performed 3D imaging of a whole brain treated with spiperone probe **3** (DRD2). As shown in Figure 6F, the distribution of probe **3** (DRD2) was clearly observed in the brain-wide level, revealing probe **3** (DRD2) was localized with remarkably high selectivity in the striatum and cerebral cortex where DRD2 and 5HT2A are expressed, respectively. The 3D distributions of PFQX probe **2** (AMPAR) and GluD2 nanobody **7** (GluD2) were also visualized, which agrees with the expression areas of the corresponding natural receptors (Figure S10). Therefore, the combination of *FixEL* and tissue clearing technologies, such as 3DISCO, enabled the evaluation of the 3D distribution of MOI in the brain from whole brain (mm) scale to dendritic spine (μm) scale.

## Discussion

By revisiting traditional PFA-mediated tissue fixation chemistry and coupling with rational molecular design, we were able to develop a new method to capture and visualize exogenously administered small molecules in the brain. *FixEL* is a rapid and simple method that does not require special instruments. *FixEL* requires chemical modification of the ligand with a primary amine and fluorescent dye, which may affect its affinity, membrane permeability, and tissue adsorption. However, MOI tested here exhibited the spatial distribution consistent with the expression areas of proteins targeted by the corresponding ligands. These results may reveal the chemical decoration of the ligand gives minimal impact on the corresponding ligand properties. In contrast with the recently reported Click-ExM^17^ and CATCH,^23^ our method is applicable to small molecules often used in medicine and physiology, which non-covalently interact with proteins. This method is highly compatible with existing analytical methods including CLSM, immunostaining, and tissue clearing technology that allows for 3D imaging. *FixEL* can provide various information of MOI probes in a live brain, including target/off-target proteins binding in various areas, time-dependent localization changes, and diffusion/retention kinetics with high spatial resolution of the whole brain. Importantly, *FixEL* can be simply extended to nanobodies. Although nanobody is now regarded as a promising biopharmaceutical, as well as powerful biotechnology tools, its spatial distribution *in vivo* has not been clearly evaluated to date. To our knowledge, this is the first report on the imaging of nanobody’s distribution and diffusion in tissue with sub μm resolution.

It was reported that the standard spatial resolution of PET imaging techniques is around the size of organ structures (1–10 mm size), and no synapse-level resolution data are available to date.^28,29,39,47^ In contrast, we demonstrated here that *FixEL* carried out the visualization of MOI at synaptic resolution (sub μm size), thanks to its fluorescence-based imaging. In addition, *FixEL* can be combined with the conventional immunostaining methods, leading to precise comparison of the spatial distribution of MOI and target protein. Using this benefit, we succeeded in obtaining direct evidence on the interaction of CNITM and spiperone to the corresponding target receptors (mGlu1 and DRD2/5HT2A, respectively). While we here showed the utility of *FixEL* to analyze MOI distribution in the brain, it is reasonable to envision its application to the whole body of mice. Overall, the unique features of *FixEL* should contribute to the accelerated development and fine optimization of pharmaceuticals and PET tracers, by correlation with various imaging techniques.

Indeed greatly advanced, 3D MS imaging requires a longer measurement time for thick samples, compared with *FixEL*. In our experiment, *FixEL* performed 3D imaging of MOI in the whole mouse brain with 3-μm axial resolution and 0.8 μm × 0.8 μm lateral resolution within 2 h using a light sheet microscope (Figure S11). On the other hand, it is roughly estimated, on the basis of the state-of-the-art MS equipment with high speed, that MS imaging needs about one month for a 3-μm-thick serial section of the whole mouse brain with 50 μm **×** 50 μm lateral resolution.^22^

We mainly employed ligands that interact with membrane proteins in this study, but *FixEL* is potentially applicable to MOI-targeting intracellular proteins (Figure S12A). As a proof of principle, we demonstrated the *FixEL* probe for HSP90, an intracellularly expressed chaperone protein. We conjugated an amine-tethered PU-H71, a selective inhibitor of HSP90, with BODIPY-FL instead of Ax647 (Figure S12B). The cultured cells experiments showed this probe interacted with the intracellular HSP90 and is tightly fixed by *FixEL* for imaging (Figure S12C). Also, our results shown in Figure S1 revealed that a strategy for stimuli-responsive uncaging of amine is compatible with *FixEL*, allowing chemical sensing of the environmental changes proximal to a protein-of-interest. In recent years, a variety of chemical biology probes are developed for sensing the biological environment. However, most of them are used for live cell experiments, but are not available in fixed cells or tissues, since they are easily washed away. We envision that a rational combination of such probes with *FixEL* technology may expand the scope of this methodology for *in vivo* imaging with a higher resolution for diverse targets.

## Experimental Procedures

### Resource availability

#### Lead contact

Further information and requests for resources should be directed to and will be fulfilled by the lead contact, Prof. Itaru Hamachi, Department of Synthetic Chemistry & Biological Chemistry, Graduate School of Engineering, Kyoto University, ihamachi@sbchem.kyoto-u.ac.jp.

#### Materials availability

All materials generated in this study are commercially available, can be prepared, or can be synthesized as described.

### Synthesis

All synthesis procedures and characterizations are described in the supplemental information (Figures S13-S24).

### Subcloning and preparation of a dye-conjugated GluD2 nanobody

The protocols of subcloning, preparation, and characterization of an Ax647-conjugated GluD2 nanobody are described in the supplemental information.

### Fluorescence imaging

Fluorescence imaging was performed using a CLSM (Leica Microsystems, Germany, TCS SP-8) equipped with 5× objective (numerical aperture [NA] = 0.15 dry objective), 10**×** objective (NA = 0.40 dry objective), 40**×** objective (NA = 1.30 oil objective), 63**×** objective (NA = 1.40 oil objective), 100**×** objective (NA = 1.40 oil objective), and a GaAsP detector. The excitation laser was derived from a white laser and was set to an appropriate wavelength depending on the dye. Lightning deconvolution process (LAS X 3.5.5, Leica Microsystems, Germany) was used in Figures 3C, 5D, S4D, and S5C. The procedures and conditions are described in the following figure captions and the supplemental information.

### Injection of reagents into the mouse cerebellum

Experiments were conducted according to the literature using 5 weeks old mice (male, C57BL/6N strain; body weight 18–22 g).^48^ mGlu1 KO mice were purchased from Laboratory Animal Resource Center (Tsukuba University, Japan).^49^ Under deep anesthesia, probe **1** (4.5 μL) was directly injected into the vermis of cerebellar lobules V–VIII (0.5 mm depth from the surface) using a microinjector (Nanoliter 2010, World Precision Instruments) (600 nL/min).

### Injection of reagents into mouse lateral ventricle

Experiments were conducted according to the literature using 5 weeks old mice (male, C57BL/6N strain; body weight 18–23 g).^50^ Under the deep anesthesia, the probe or nanobody solution (4.5 μL) was directly injected into the LV using a micro-injector (Nanoliter 2010, World Precision Instruments) (600 nL/min).

### Animal experiments

C57BL6/N mice were purchased from Japan SLC (Shizuoka, Japan). The animals were housed in a controlled environment (23°C, 12 h light/dark cycle) and had free access to food and water, according to the regulations of the Guidance for Proper Conduct of Animal Experiments by the Ministry of Education, Culture, Sports, Science, and Technology of Japan. All experimental procedures were performed in accordance with the National Institute of Health Guide for the Care and Use of Laboratory Animals and were approved by the Institutional Animal Use Committees of Kyoto University.

## Supplementary Material

Document S1. Supplemental experimental procedures, Figures S1–S24, and supplemental references.

## Data Availability

The authors declare that the data supporting the findings of this study are available with the paper and its supplemental information files. The data that support the findings of this study are available from the corresponding author upon reasonable request.

## Highlights

A new imaging method for exogenously administered molecules of interest in the brain

Chemical strategy developed by revisiting the traditional tissue fixation chemistry

Visualizing the distribution changes of molecules of interest in 2D/3D mode

## The Bigger Picture

Pharmaceuticals and imaging probes for medical diagnosis/treatment are used by administering those molecules from outside the body. Precise assessment of the molecular distribution and interaction of these exogenously administered molecules of interest (eMOIs) in the body should be critical for the development/optimization of the functional molecules. However, it has been difficult to monitor the distribution dynamics of eMOIs in 3D-mode of biological tissues such as the brain. In this paper, we successfully developed a new method to capture and visualize eMOIs in the mouse brain by revisiting the traditional tissue fixation chemistry that is combined with a chemical twist. The method, named *FixEL*, allowed us to evaluate the selective/nonselective interactions to proteins, time-dependent localization changes, and diffusion/retention kinetics of eMOIs. The unique features of *FixEL* can contribute to the development and fine optimization of functional small molecules that work *in vivo*.
