# Supplementary material for: Revisiting PFA-mediated tissue fixation chemistry: FixEL enables trapping of small molecules in the brain to visualize their distribution changes
Source: Chem. Author manuscript; Available in PMC 2023 Dec 13. (PMC7615374; doi:10.1016/j.chempr.2022.11.005)
Supplement: Document S1. Supplemental experimental procedures, Figures S1–S24, and supplemental references. [file EMS192557-supplement-Document_S1__Supplemental_experimental_procedures__Figures_S1_S24__and_supplemental_references_.pdf]

Chem, Volume *9*

Supplemental information

Revisiting PFA-mediated tissue fixation chemistry: *FixEL* enables trapping of small molecules in the brain to visualize their distribution changes

Hiroshi Nonaka, Takeharu Mino, Seiji Sakamoto, Jae Hoon Oh, Yu Watanabe, Mamoru Ishikawa, Akihiro Tsushima, Kazuma Amaike, Shigeki Kiyonaka, Tomonori Tamura, A. Radu Aricescu, Wataru Kakegawa, Eriko Miura, Michisuke Yuzaki, and Itaru Hamachi

Supplemental Figures

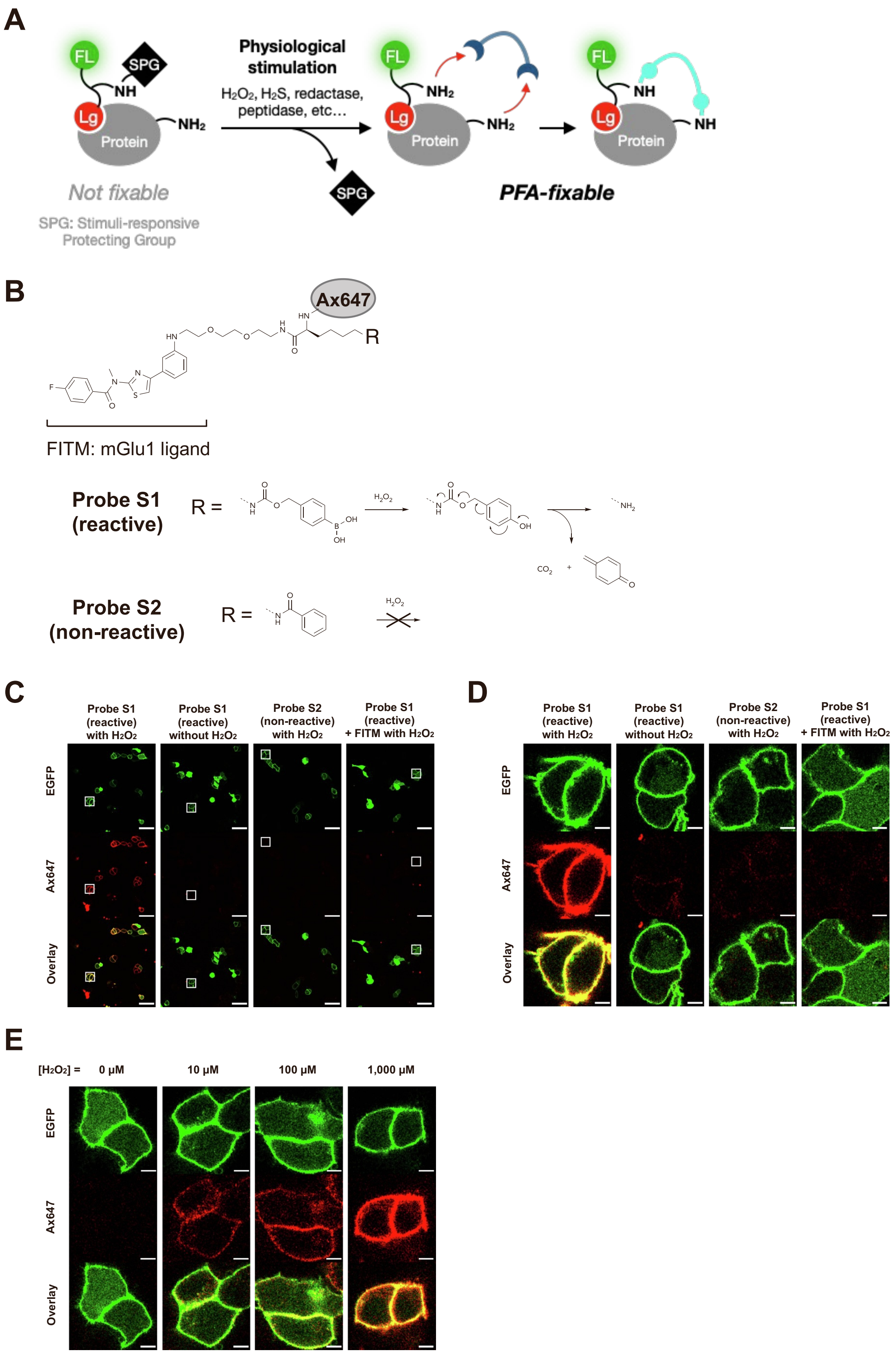

Figure S1. Stimuli-responsive chemical biology probe by using *FixEL*

(A) Schematic illustration of stimuli-responsive *FixEL* probe.

(B) Chemical structures of H_2O_2-responsive *FixEL* probe **S1** and control compound **S2**.

(C, D) CLSM imaging of mGlu1 expressed on HEK293T cells treated with *FixEL* probe **S1**, **S1** with 20 eq. of FITM, or probe **S2** (Ax647, red) in the presence or absence of 100 μM H_2O_2. EGFP-F was used as a transfection marker (Green).

(E) Confocal imaging of mGlu1 and EGFP-F (green) expressed on HEK293T cells treated with a *FixEL* probe **S1** (Ax647, red) upon addition of 0, 10, 100, or 1000 μM H_2O_2. Fluorescence imaging of the cells was performed using a CLSM equipped with a 40× objective and a GaAsP detector (488 nm excitation for EGFP-F and 633 nm excitation for Ax647). Scale bar 5 μm.

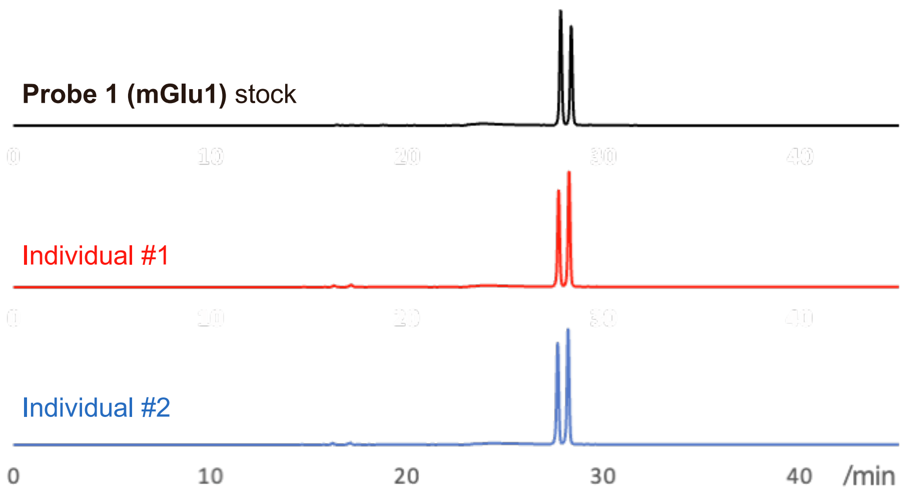

Figure S2. HPLC analysis of the extract from probe-administered mouse cerebellum

20 μM of probe **1** (mGlu1) was injected into the mouse cerebellum. After 6 h incubation, the whole cerebellum was isolated and the homogenate was extracted with methanol/H_2O. After centrifugation, the supernatant was analyzed by RP-HPLC. RP-HPLC analysis was conducted on a Shimadzu Nexera system equipped with a fluorescent detector (640/660 nm (Ex/Em)) with a linear gradient of 0-40 % CH_3CN/10 mM NH_4OAc aq. (40 min) and 40-100% (5 min). Probe **1** (mGlu1) exhibited two peaks on the HPLC chromatogram derived from diastereomeric isoforms.

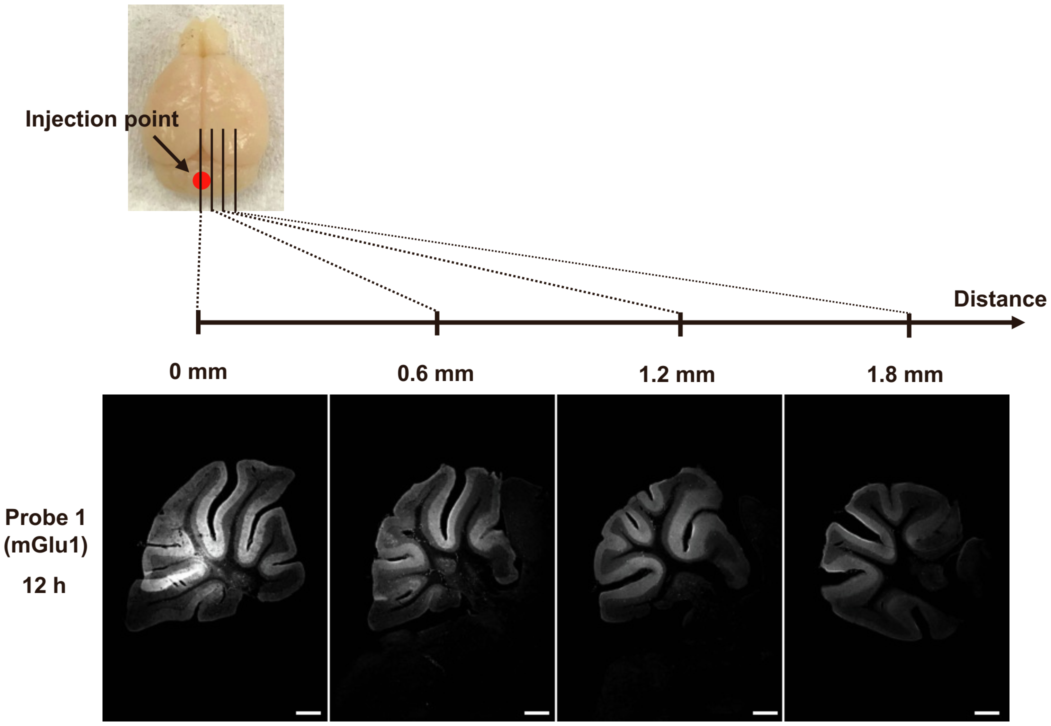

Figure S3. Fluorescence imaging of cerebellum slices after *FixEL* with probe 1 (mGlu1) at different distances from the midline of the cerebellum, related to Figure 3

Fluorescence imaging of cerebellum slices after *FixEL* with probe **1** (mGlu1). PBS(–) containing 20 μM of probe **1** (mGlu1) (4.5 μL) was injected into the mouse cerebellum. After 12 h of incubation, the mouse was transcardially perfused with 4% PFA. After slice preparation (40-μm thick), fluorescence imaging of the slices was performed using a CLSM equipped with a 5× objective and a GaAsP detector (633 nm excitation for Ax647). Scale bar: 500 μm.

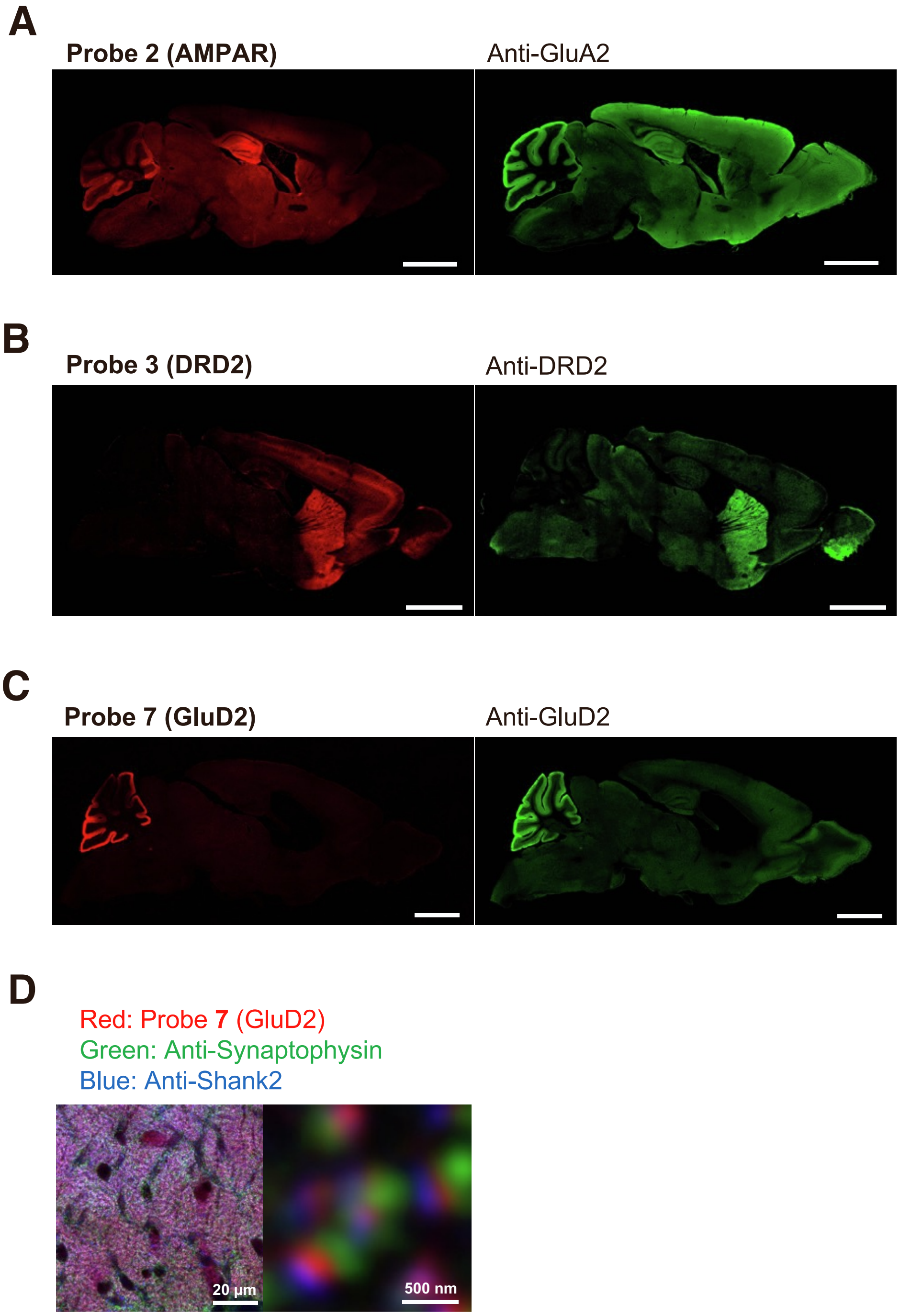

Figure S4. Fluorescence imaging of whole-brain sagittal slices after *FixEL* using probe 2 (AMPAR), 3 (DRD2), and 7 (GluD2), related to Figure 5

(A) Co-immunostaining of whole-brain sagittal slices after *FixEL* with probe **2** (AMPAR). PBS(−) containing 40 μM of probe **2** (AMPAR) (4.5 μL) was injected into the mouse lateral ventricle. After 3 h of incubation, the mouse was transcardially perfused with 4% PFA. The slices (15-μm thick)

after *FixEL* with probe **2** (AMPAR) were treated with heat-induced-epitope-retrieval process with ImmunoSaver (FUJIFILM Wako: 80ºC 20 min) and immunostained using anti-GluA2 (Merck, MAB397). GluA2 is one of the AMPA receptor subunits. Fluorescence imaging was performed using a CLSM equipped with a 10× objective and a GaAsP detector (594 nm excitation for Ax594, 647 nm excitation for Ax647). Scale bar: 2 mm.

(B) Co-immunostaining of whole-brain sagittal slices after *FixEL* with probe **3** (DRD2). PBS(−) containing 25 μM of probe **3** (DRD2) (4.5 μL) was injected into the mouse lateral ventricle. After 8 h of incubation, the mouse was transcardially perfused with 4% PFA. The slices (50-μm thick) after *FixEL* with probe **3** (DRD2) were permeabilized and immunostained using anti-DRD2 (Frontier Institute, D2R-Rb-Af960). Fluorescence imaging was performed using a CLSM equipped with a 5× objective and a GaAsP detector (488 nm excitation for Ax488, 633 nm excitation for Ax647). Scale bar: 2 mm.

(C) Co-immunostaining of whole-brain sagittal slices after *FixEL* with probe **7** (GluD2). PBS(−) containing 10 μM of probe **7** (GluD2) (4.5 μL) was injected into the mouse lateral ventricle (left and right). After 24 h of incubation, the mouse was transcardially perfused with 4% PFA. The slices (50-μm thick) after *FixEL* with probe **7** (GluD2) were permeabilized and immunostained using anti-GluD2 (SIGMA, HPA056253). Fluorescence imaging was performed using a CLSM equipped with a 10× objective and a GaAsP detector (488 nm excitation for Ax488, 633 nm excitation for Ax647). Scale bar: 2 mm.

(D) Co-immunostaining of the sagittal slices with *FixEL* probe **7** (GluD2). The slices after *FixEL* were permeabilized and immunostained using anti-synaptophysin (green, Ax488-conjugated secondary antibody) and anti-Shank2 (blue, Ax405-conjugated secondary antibody). Fluorescence imaging was performed using a CLSM equipped with a 100× objective, a GaAsP detector (405 nm excitation for Ax405, 488 nm excitation for Ax488, and 633 nm excitation for Ax647), and Lightning deconvolution.

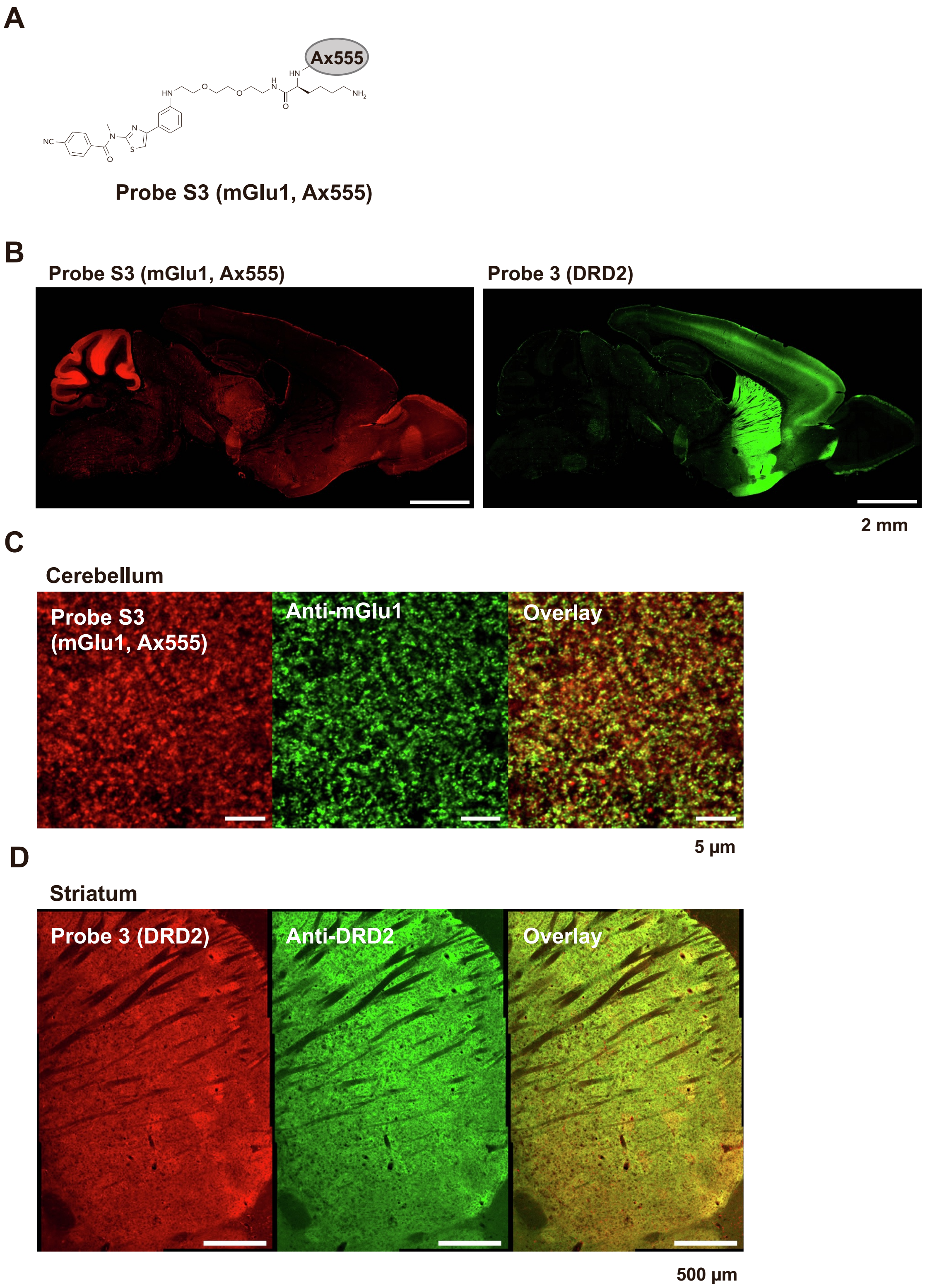

Figure S5. Dual-color imaging of mGlu1 and DRD2 ligand in a single mouse

(A) Chemical structure of probe **S3**.

(B) Fluorescence imaging of a single whole brain slice with *FixEL* of mGlu1 ligand (Ax555, probe **S3**; 6h after LV injection) and DRD2 ligand (Ax647, probe **3**; 20h after LV injection). Imaging was performed using a CLSM equipped with a 10× objective and a GaAsP detector (561 nm and 633 nm excitation for Ax555 and Ax647, respectively). Scale bar: 2 mm.

(C) Co-immunostaining of the brain slice with anti-mGlu1. *FixEL* of mGlu1 ligand (Ax555) and anti-mGlu1 are shown in red and green, respectively. A fluorescence image was acquired by using a CLSM equipped with a 100× objective, a GaAsP detector, and Lightning deconvolution. Scale bar: 5 μm.

(D) Co-immunostaining of the brain slice with anti-DRD2. *FixEL* of DRD2 ligand (Ax647) and anti-DRD2 are shown in red and green, respectively. The fluorescence image was acquired by using a CLSM equipped with a 10× objective and a GaAsP detector. Scale bar: 500 μm.

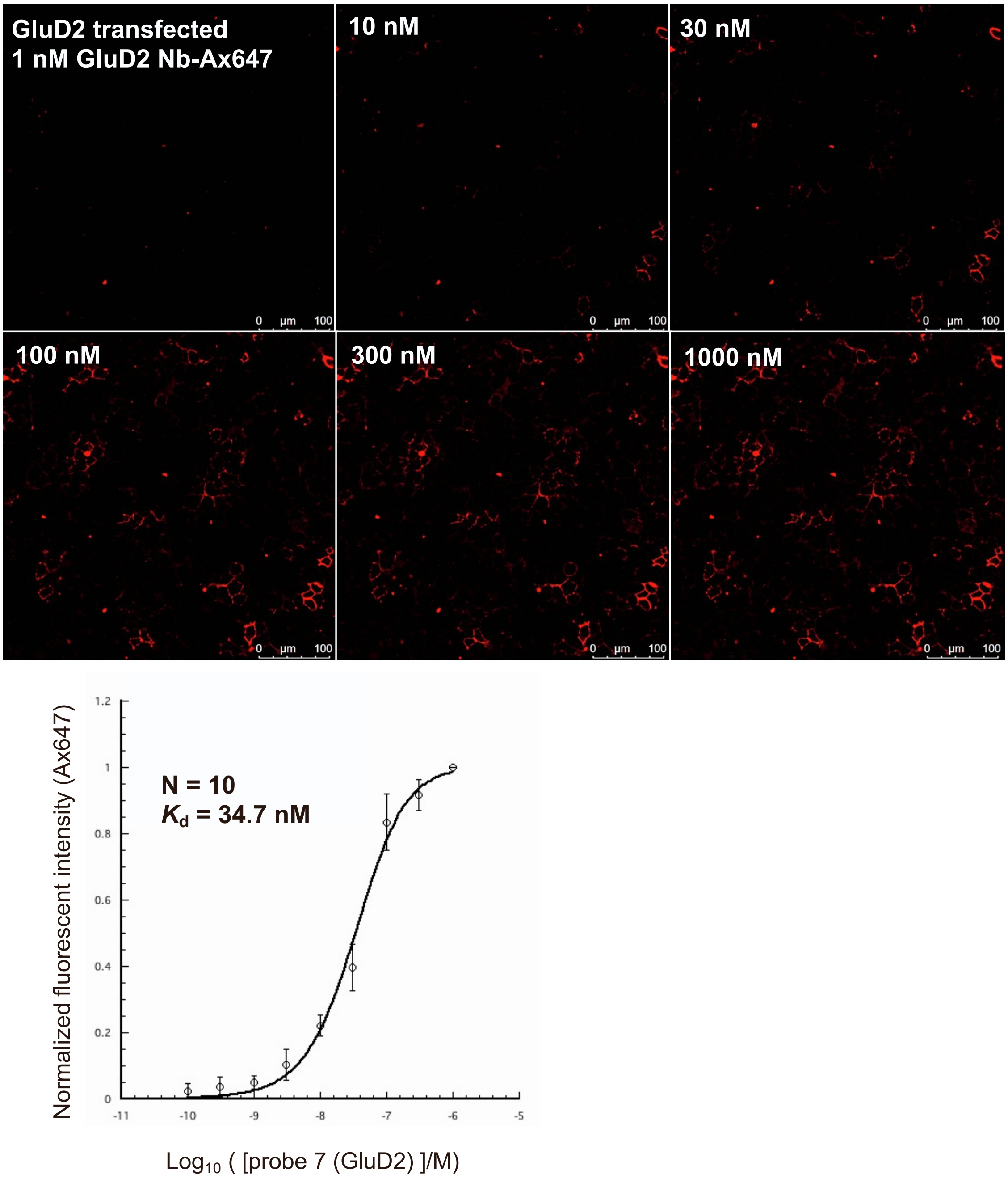

Figure S6. Determination of probe 7 (GluD2) affinity

Fluorescence change of the plasma membrane on GluD2-expressed HEK293T cells with increasing the concentration of probe **7** (GluD2). N = 10 cells in a single dish, Data are presented as mean ± s.e.m.

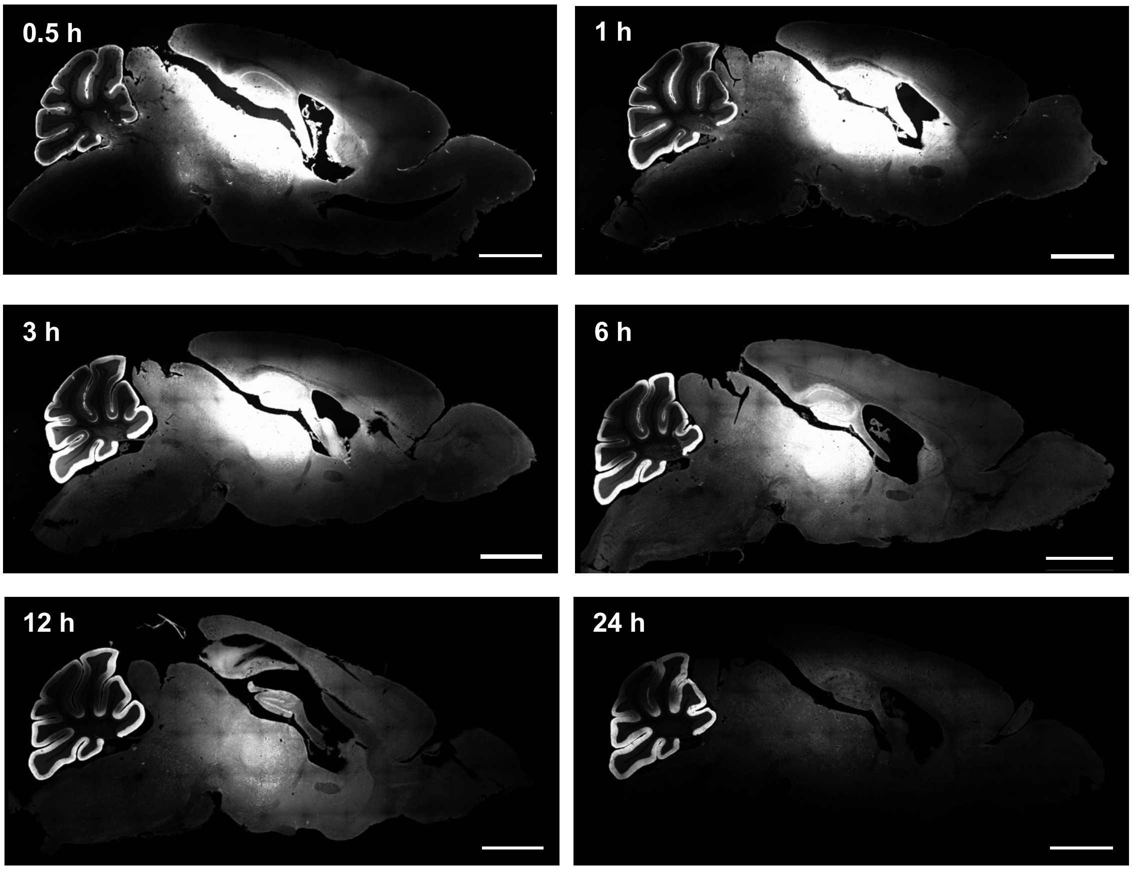

Figure S7. Visualization of distribution changes of probe 7 (GluD2) after *FixEL* at different incubation time, related to Figure 5

PBS(−) containing 40 μM of probe **7** (GluD2) (4.5 μL) was injected into the mouse lateral ventricle. After 0.5, 1, 3, 6, 12, or 24 h of incubation, the mouse was transcardially perfused with 4% PFA. Fluorescence imaging of slices (50-μm thick) was performed using a CLSM equipped with a 5× objective and a GaAsP detector (633 nm excitation for Ax647). Scale bar: 2 mm.

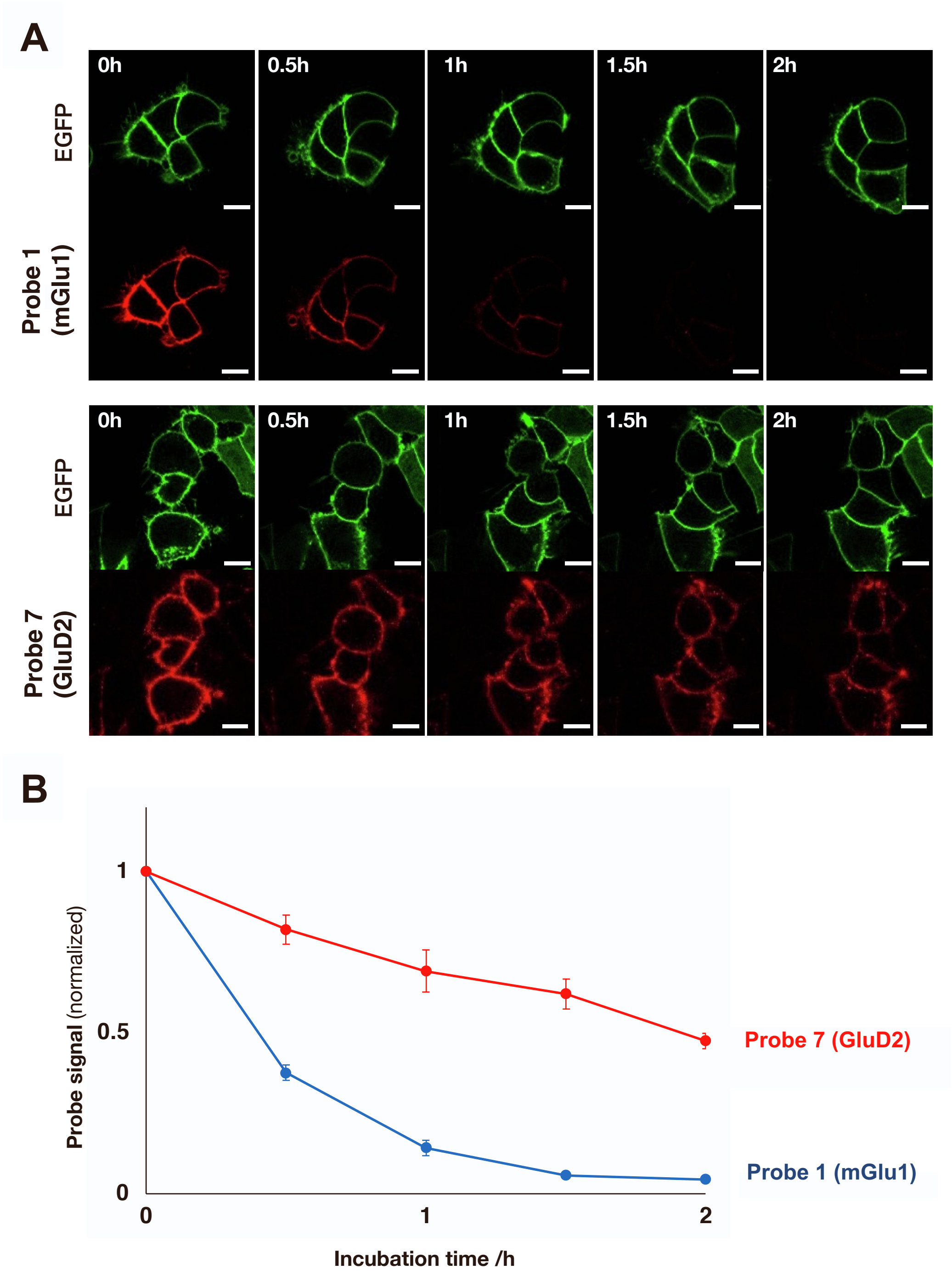

Figure S8. Comparison of the dissociation rate between probe 1 (mGlu1) and 7 (GluD2) in living cells

(A) Confocal imaging of mGlu1 or GluD2 and EGFP-F (green) expressed HEK293T cells after adding probe **1** (mGlu1) or **7** (GluD2) (Ax647, red), washing with PBS(–), and incubation with DMEM in a 5% CO_2 humidified chamber at 37 °C for 0, 0.5, 1, 1.5, and 2h. Fluorescence imaging

of the cells was performed using a CLSM equipped with a 40× objective and a GaAsP detector (488 nm excitation for EGFP and 633 nm excitation for Ax647). Scale bar 10 μm.

(B) Fluorescence change of the plasma membrane on mGlu1 or GluD2-expressed HEK293T cells depending on the incubation time with probe **1** (mGlu1) (blue circle) or probe **7** (GluD2) (red square). n = 8 cells. Data are presented as mean ± s.e.m. Fluorescent signal of probe **7** (GluD2) from cell membrane still presented even after 2 hours of incubation, while probe **1** (mGlu1) signal was almost disappeared after 1.5 hour of incubation.

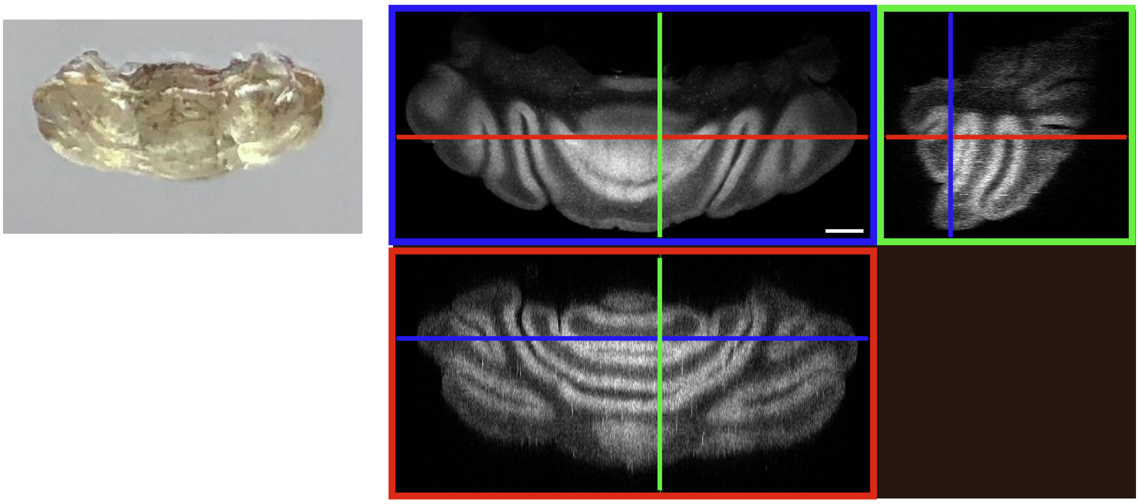

Figure S9. 3D imaging of cerebellum regions after *FixEL* with probe 1 (mGlu1) and 3DISCO, related to Figure 6

PBS(–) containing 20 μM of probe **1** (mGlu1) (4.5 μL) was injected into the mouse cerebellum. After 12 h of incubation, the mouse was transcardially perfused with 4% PFA. After 3DISCO treating, z-stacking fluorescence imaging of the cerebellum was performed using a CLSM equipped with a 5× objective and a GaAsP detector (633 nm excitation for Ax647). Scale bar: 500 μm.

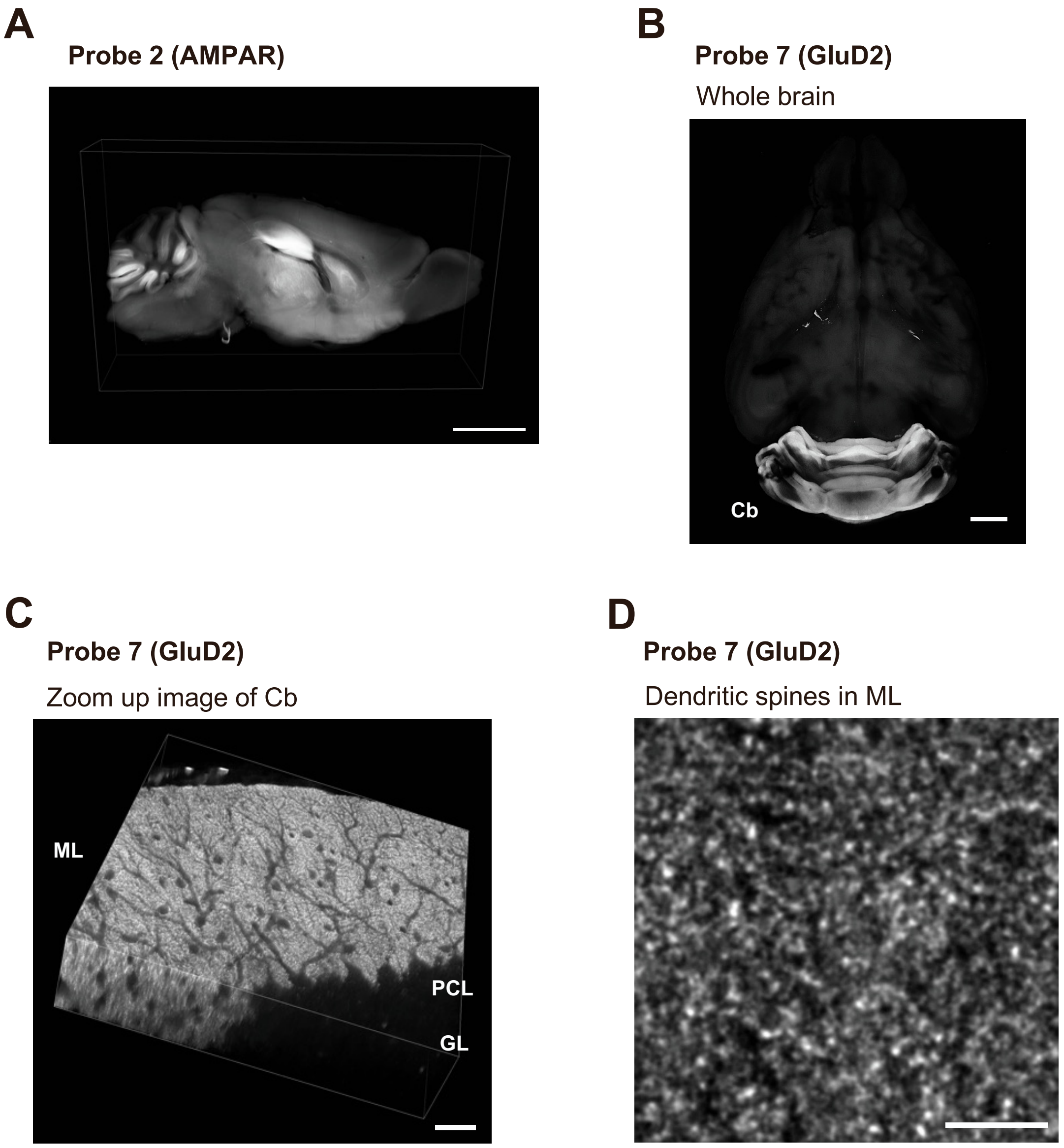

Figure S10. 3D imaging of the brains after *FixEL* and 3DISCO tissue clearing, related to Figure 6

(A) Z-stacking fluorescence imaging of *FixEL* probe **2** (AMPAR). PBS(–) containing 40 μM of probe **2** (AMPAR) (4.5 μL) was injected into mouse lateral ventricle. After 3 h of incubation, the mouse was transcardially perfused with 4% PFA. After 3DISCO treating, z-stacking fluorescence imaging of the whole brain was performed using a CLSM equipped with a 5× objective and a GaAsP detector (633 nm excitation for Ax647). Scale bar: 2 mm.

(B) Z-stacking fluorescence imaging of *FixEL* probe **7** (GluD2). PBS(–) containing 25 μM of probe **7** (GluD2) (4.5 μL) was injected into mouse lateral ventricle (left and right). After 20 h of incubation,

the mouse was transcardially perfused with 4% PFA. After 3DISCO treating, z-stacking fluorescence imaging of the whole brain was performed using a CLSM equipped with a 5× objective and a GaAsP detector (633 nm excitation for Ax647). Scale bar: 2 mm.

(C) Z-stacking fluorescence imaging of cerebellum region in Figure S10B with a 40× objective. Scale bar: 20 μm.

(D) Enlarged image of the cerebellar molecular layer (ML) in Figure S10C. Scale bar: 5 μm.

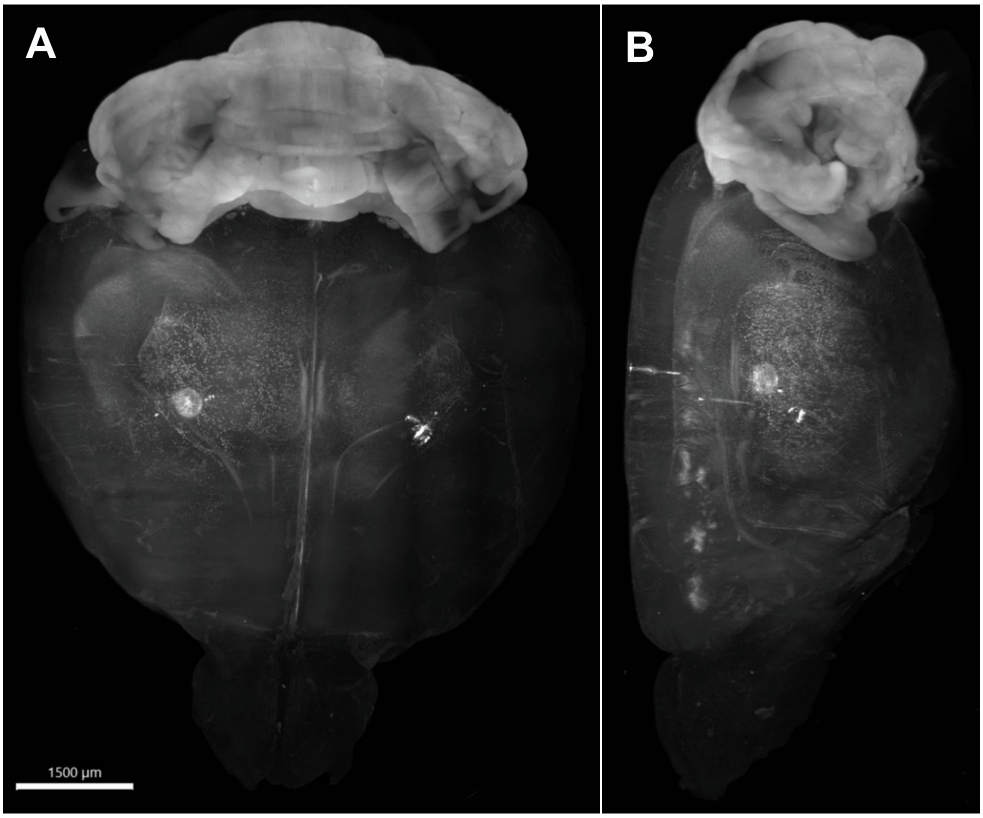

Figure S11. High-resolution 3D fluorescence imaging of *FixEL* sample by light sheet fluorescence microscopy, related to Figure 6

3D rendering of the whole brain distribution of probe **7** (GluD2) in the top (A) and side (B) view. PBS(–) containing 25 µM of probe **7** (GluD2) (4.5 µL) was injected into the mouse lateral ventricle (left and right). After 20 h of incubation, the mouse was transcardially perfused with 4% PFA. After 3DISCO treatment, z-stacking fluorescence imaging of the whole brain was performed using a light sheet fluorescence microscopy (MuVi SPIM CS, Luxendo) equipped with a 10× objective (642 nm excitation for Ax647). The authors thank Mr. Soga (AIRIX corp.) for taking the image. Scale bar: 1.5 mm.

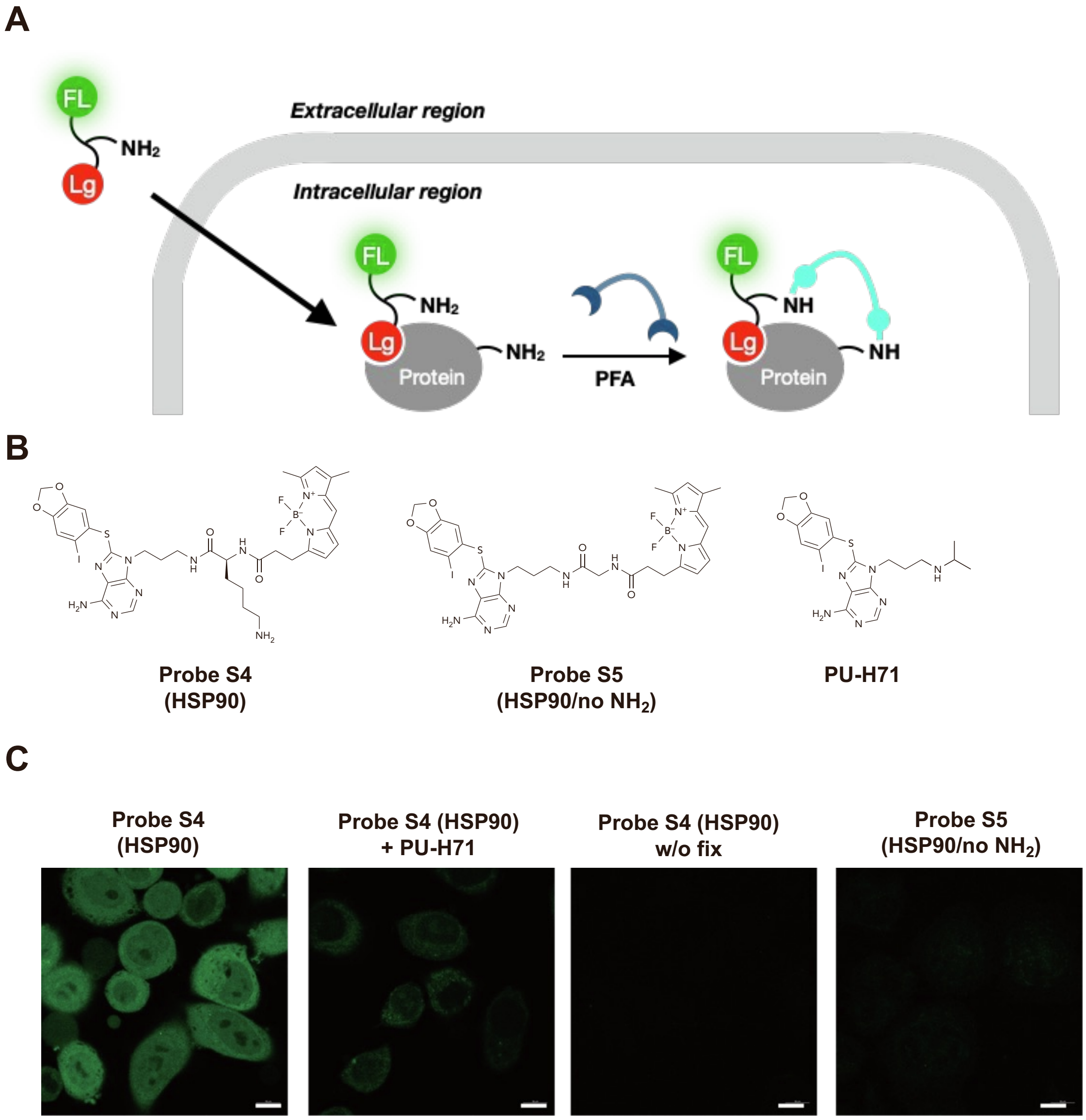

Figure S12. *FixEL* applied to HSP90 endogenously expressed in SK-BR-3 cells

(A) Schematic illustration of *FixEL* probe for targeting intracellular protein.

(B) Chemical structures of probe **S4**, **S5**, and a competitive ligand, PU-H71 targeting endogenously expressed HSP90.

(C) Confocal imaging of HSP90 endogenously expressed in SK-BR-3 cells after treatment with probe **S4** (100 nM), probe **S4** in the presence of PU-H71 (10 μM, 100 eq.), probe **S4** without PFA fixation, and probe **S5** (100 nM) (BODIPY-FL, green). Fluorescence imaging of the cells was conducted using a CLSM equipped with a 63× objective and a GaAsP detector (488 nm excitation). Scale bar 10 μm. The cells were incubated with a probe (100 nM) in PBS at 25 °C for 10 min before the fixation with 2% PFA/PBS at 25 °C for 30 min. Then, the cells were

incubated in McCoy's 5A (modified) medium (Thermo Fisher Scientific) containing 10% FBS at 37 °C overnight to remove non-fixed probes.

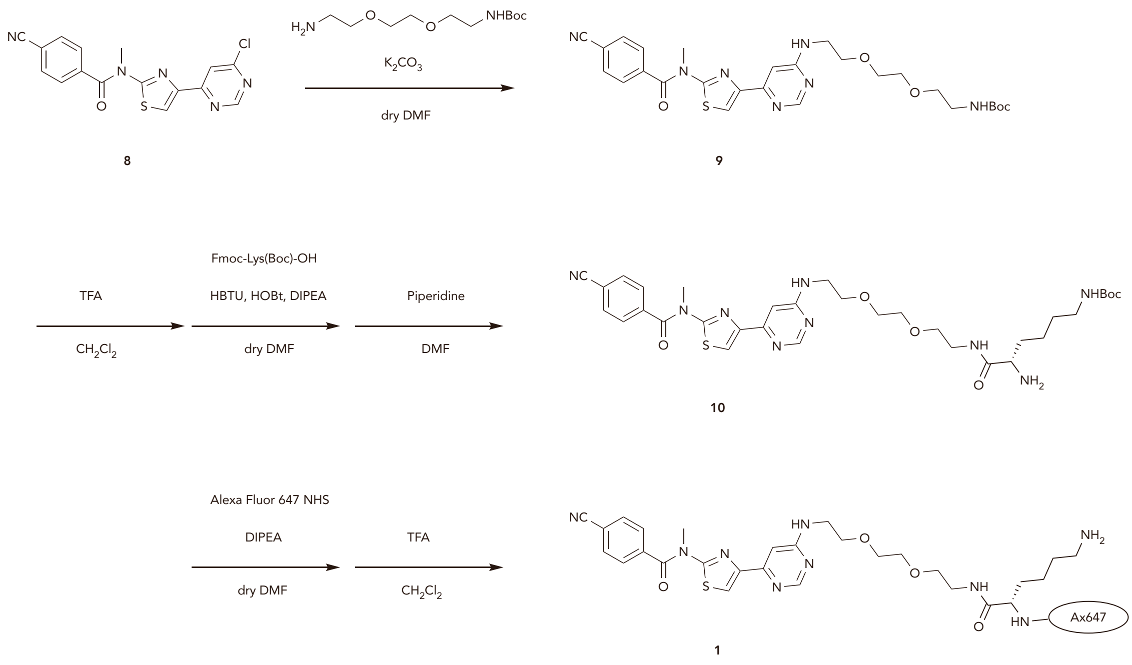

Figure S13. Synthesis of probe 1

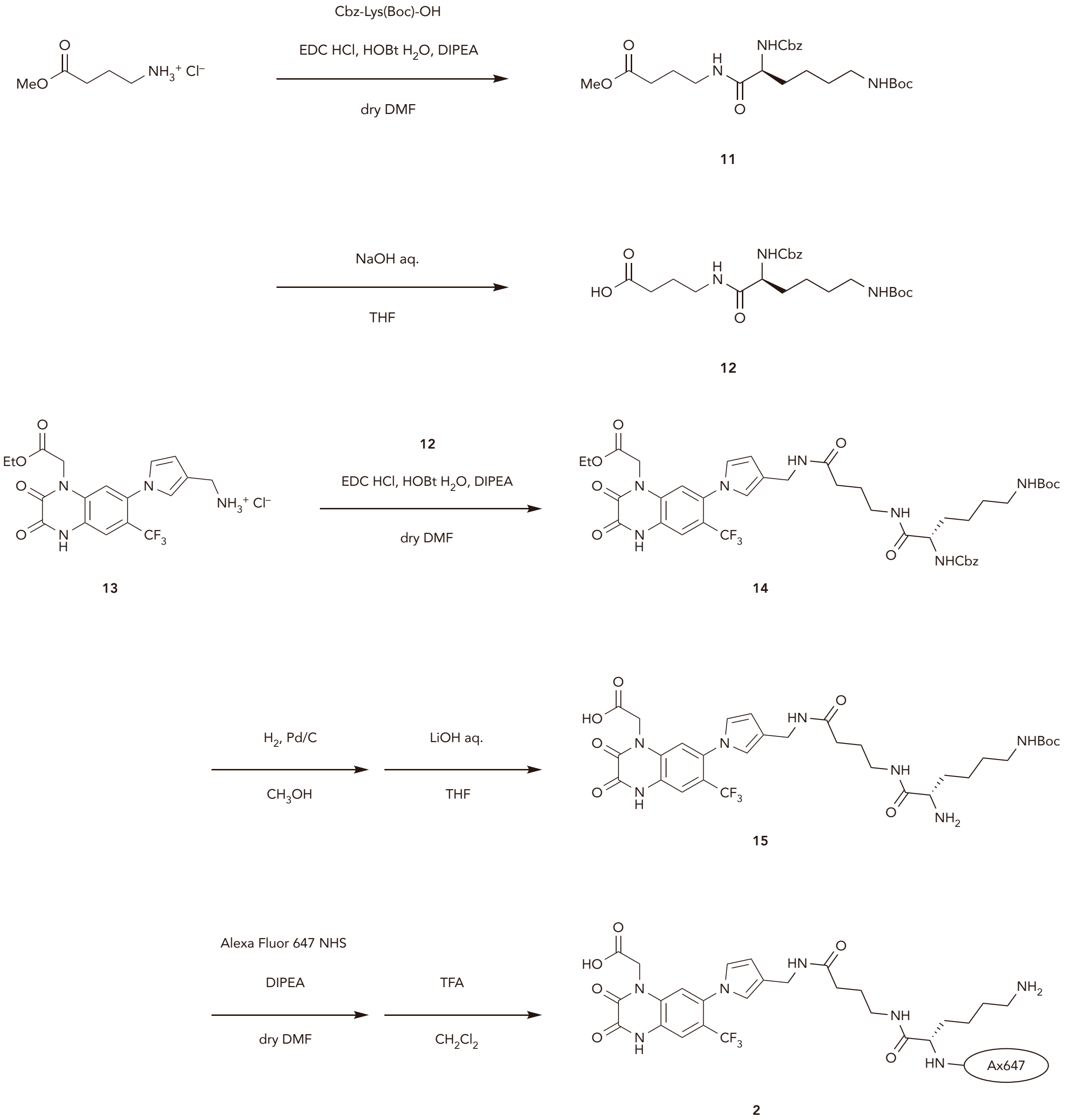

Figure S14. Synthesis of probe 2

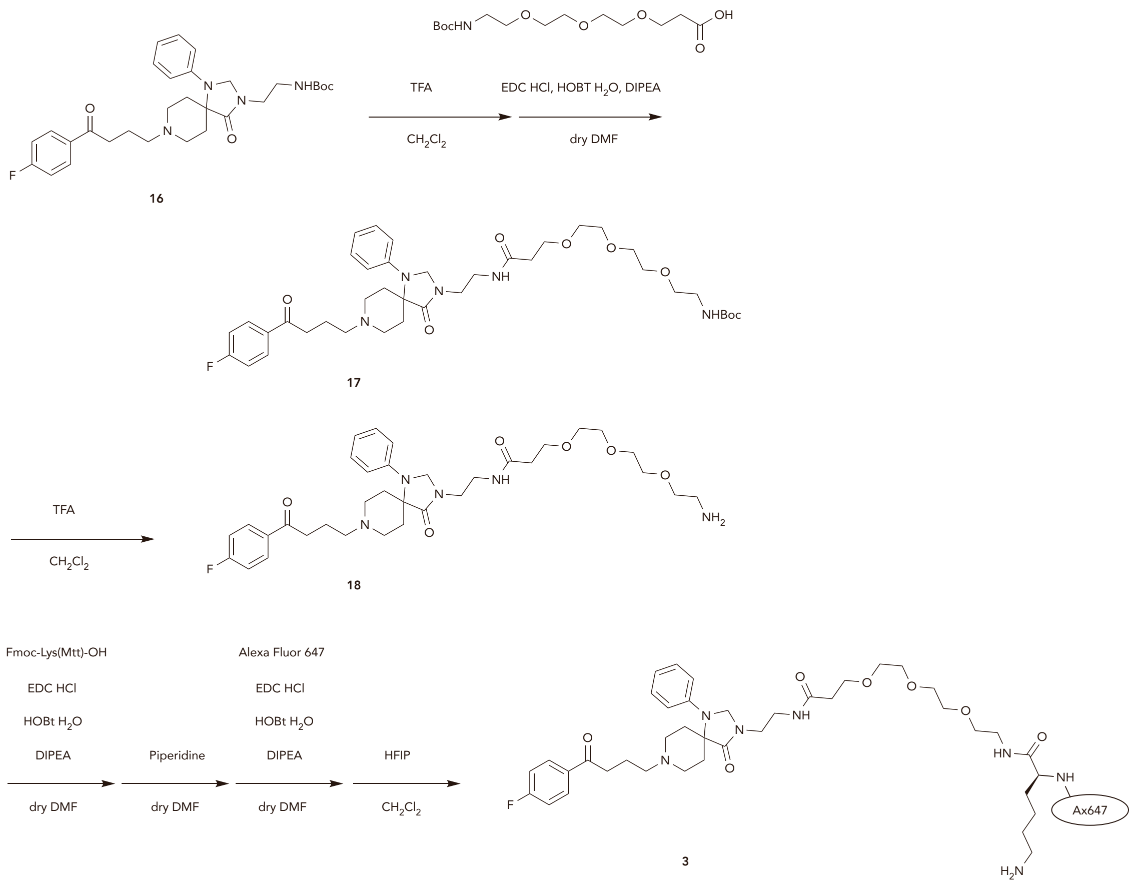

Figure S15. Synthesis of probe 3

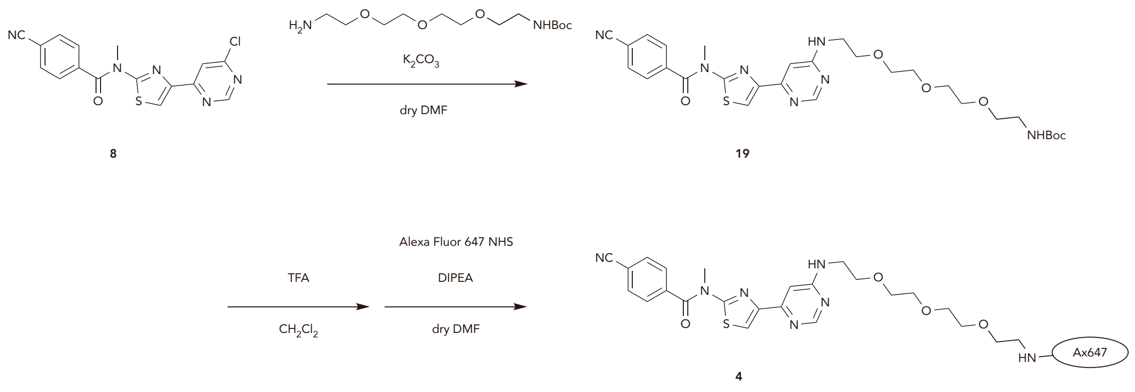

8, 19, 4

Figure S16. Synthesis of probe 4

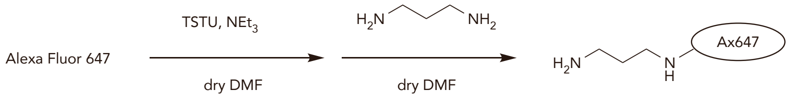

Figure S17. Synthesis of probe 5

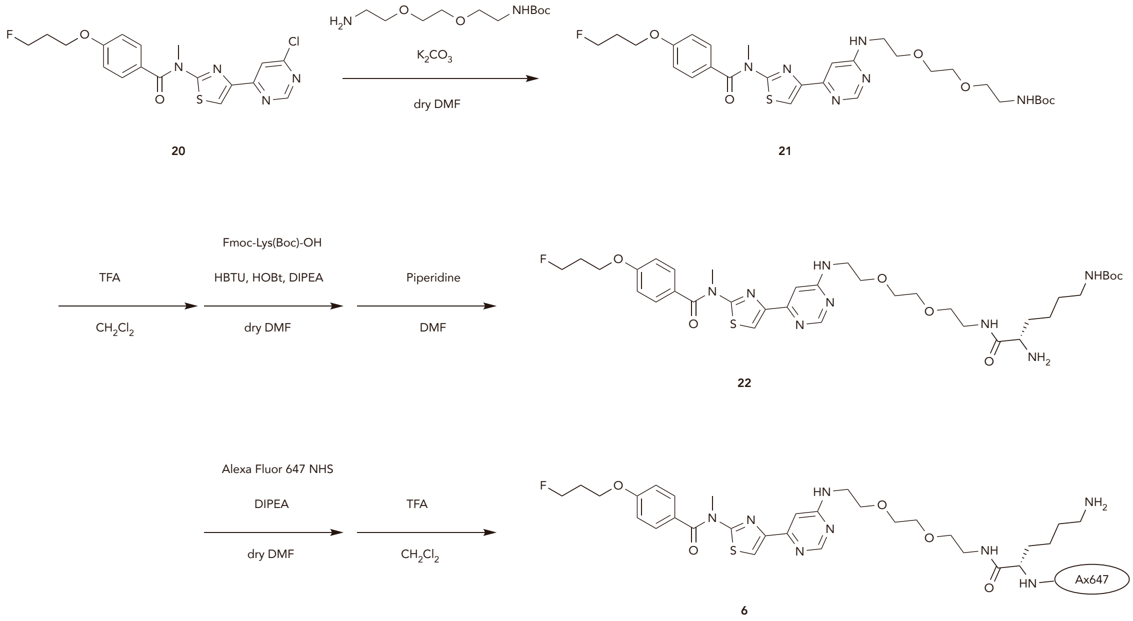

Figure S18. Synthesis of probe 6

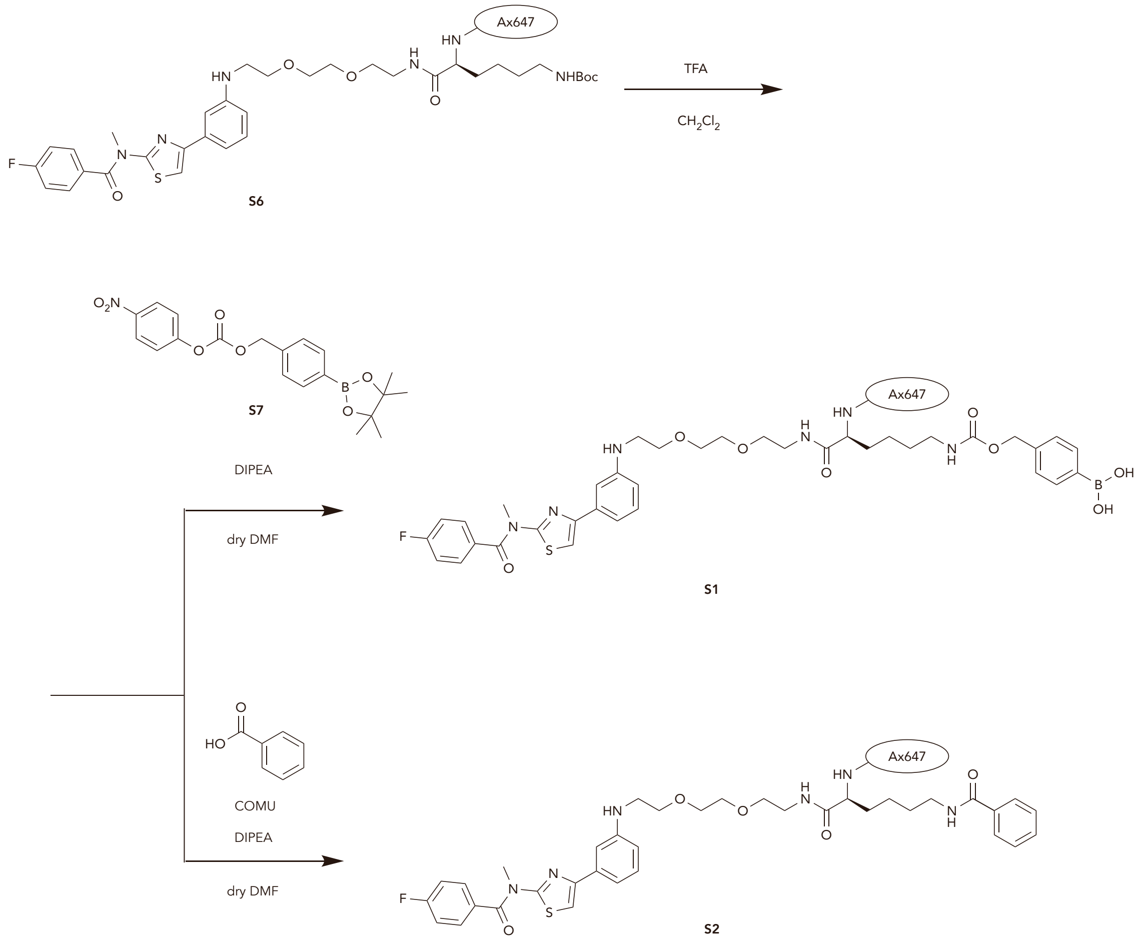

Figure S19. Synthesis of probe S1 and S2

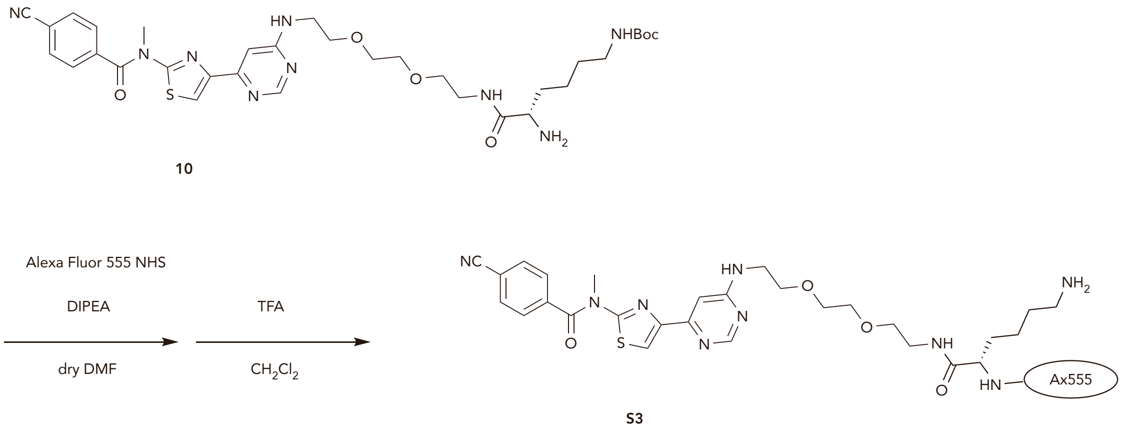

Figure S20. Synthesis of probe S3

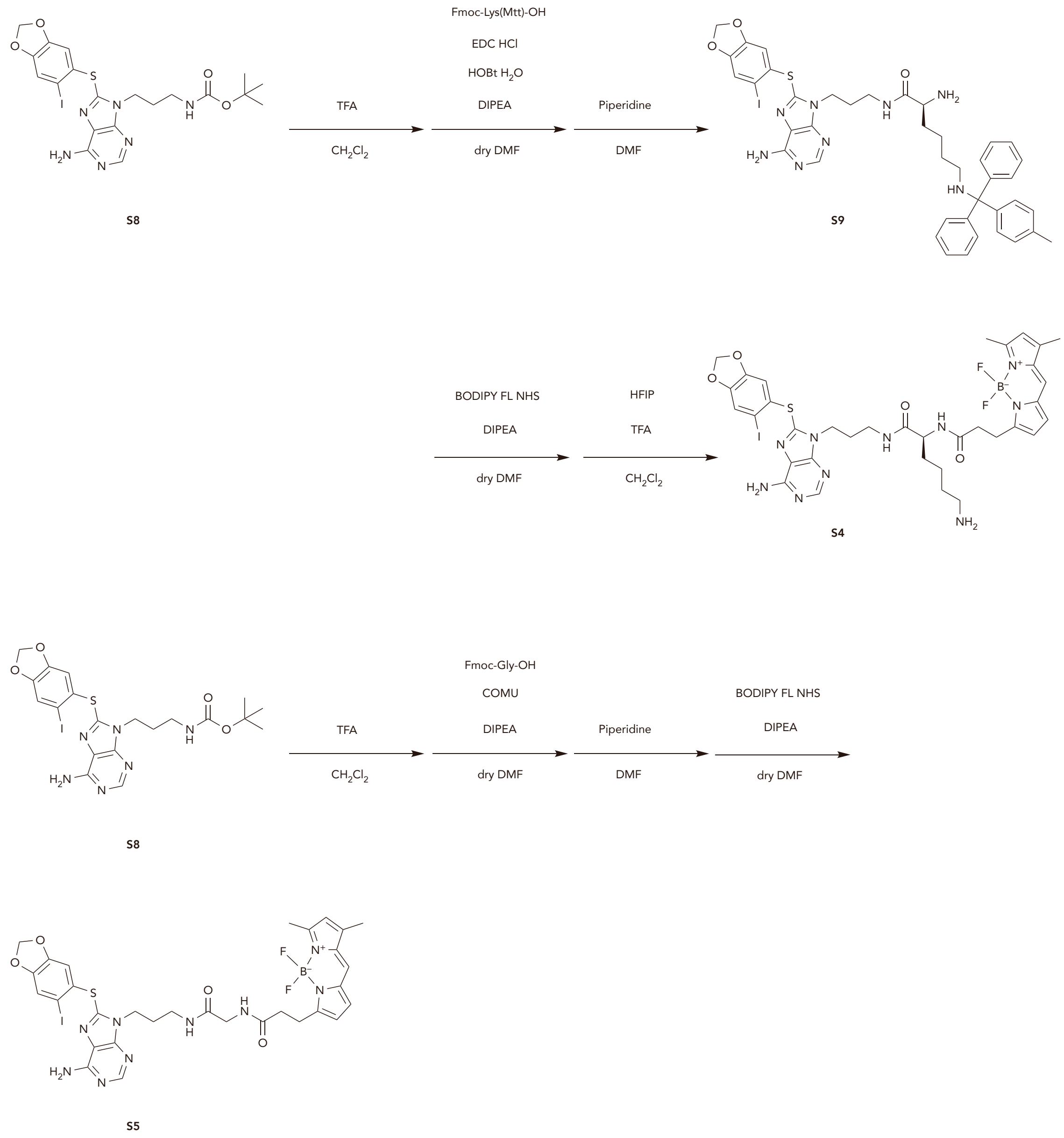

Figure S21. Synthesis of probe S4 and S5

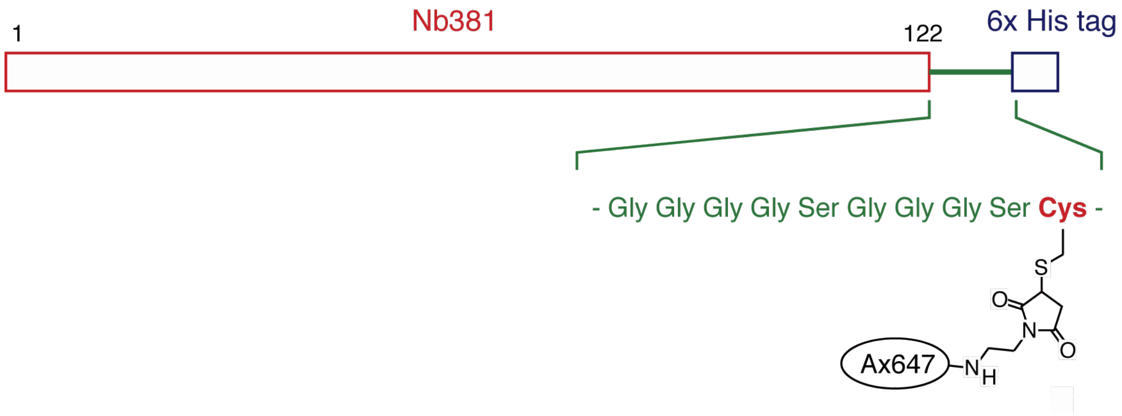

Figure S22. Structure of Nb381-Ax647 (probe 7 (GluD2))

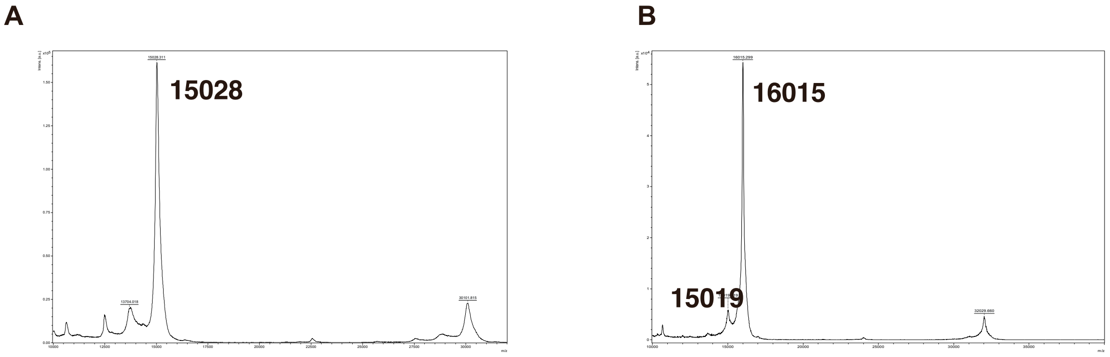

Figure S23. MALDI TOF MS spectra of Nb381-(GS)2-Cys (A) and Nb381-Ax647 (B)

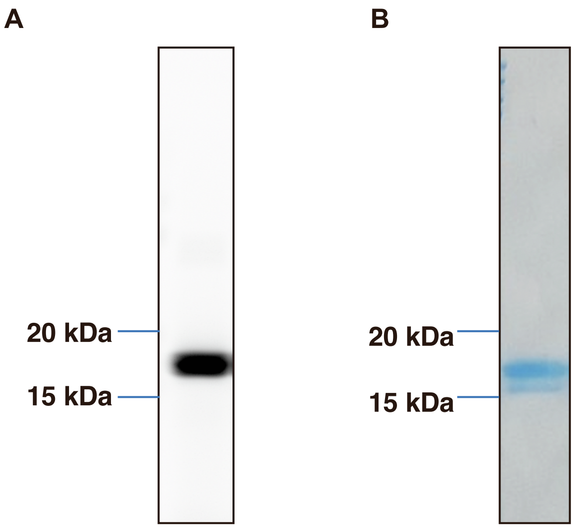

Figure S24. SDS-PAGE analysis of Nb381-Ax647

In Gel fluorescence (A) and CBB staining (B) images of Nb381-Ax647. 15% acryl amide gel. The modification yield was estimated to be 80% based on the band intensities of CBB-staining bands.

Supplemental Experimental Procedures

Synthesis and Characterization of Compounds

General materials and methods for organic synthesis

All chemical reagents and solvents were obtained from commercial suppliers (Aldrich, Tokyo Chemical Industry (TCI), Wako Pure Chemical Industries, or Watanabe Chemical Industries) and used without further purification. ^{1}H-NMR spectra were recorded in deuterated solvents on a JEOL ECS400 or JEOL ECZ600R. Chemical shifts were referenced to residual solvent peaks or tetramethylsilane (δ = 0 ppm). Multiplicities are abbreviated as follows: s = singlet, d = doublet, t = triplet, m = multiplet. MALDI-TOF Mass spectra were measured on UltrafleXtreme (Bruker Daltonics). High resolution mass spectra were measured on an Exactive (Thermo Scientific) equipped with electron spray ionization (ESI). Reversed-phase HPLC (RP-HPLC) was carried out on a Hitachi Chromaster system equipped with a diode array.

Synthesis of 1

tert-butyl (2-(2-(2-((6-(2-(4-cyano-N-methylbenzamido)thiazol-4-yl)pyrimidin-4-yl)amino) ethoxy)ethoxy)ethyl)carbamate (**9**): The compound **8** was synthesized according to the literature.[S1] To a solution of compound **8** (16 mg, 45 μmol) in DMF (0.3 mL), *tert*-butyl (2-(2-(2-aminoethoxy)ethoxy)ethyl)carbamate (55.8 mg, 225 μmol, 5.0 eq.) and K_2CO_3 (37.5 mg, 270 μmol, 6.0 eq.) were added. The reaction mixture was stirred for 6.5 h at 50 °C. The solvent was evaporated and the residue was purified by silica gel column chromatography ($CHCl_3$:MeOH = 9:1) to yield compound **9** (11 mg, 19 μmol, 43%) as a yellow oil. ^{1}H NMR (400 MHz, $CDCl_3$): δ 8.57 (s, 1H), 7.94 (s, 1H), 7.80 (d, *J* = 8.4 Hz, 2H), 7.67 (d, *J* = 8.4 Hz, 2H), 7.07 (s, 1H), 3.71–3.63 (m, 11H), 3.55 (t, *J* = 5.2 Hz, 2H), 3.33 (m, 2H), 1.41 (s, 9H). ^{13}C NMR (150 MHz, $CDCl_3$): δ 168.43, 163.30, 159.90, 158.61, 157.31, 156.13, 148.35, 138.71, 132.61, 128.23, 117.71, 116.30, 114.91, 100.18, 79.34, 70.53-69.62, 41.06, 40.53, 38.13, 28.48. HR-ESI-MS: calcd. for $C_{27}H_{33}N_7O_5S$ $[M+Na]^+$ = 590.2156, obsd = 590.2150.

tert-butyl (*S*)-(5-amino-6-((2-(2-(2-((6-(2-(4-cyano-N-methylbenzamido)thiazol-4-yl)pyrimidin-4-yl)amino)ethoxy)ethoxy)ethyl)amino)-6-oxohexyl)carbamate (**10**): To a stirred solution of compound **9** (3.0 mg, 6.4 μmol) in CH_2Cl_2 (0.5 mL), trifluoroacetic acid (TFA) (0.5 mL) was added and the reaction solution was stirred at RT for 30 min. After azeotropic removal of TFA with toluene (2 mL x 2), the residue was dissolved in dry DMF (100 μL). Then, Fmoc-Lys(Boc)-OH

(4.5 mg, 9.6 μmol, 1.5 eq.), HBTU (7.3 mg, 19 μmol, 3.0 eq.), HOBt (3.0 mg, 20 μmol, 3.1 eq.) and DIPEA (2.6 μL, 58 μmol, 9.0 eq.) were added. After stirring the reaction mixture at RT for 2 h, piperidine (10 μL) was added and the solution was stirred at RT for another 10 min. The solvent was evaporated and the residue was purified by reversed-phase HPLC with a linear gradient of 30-60 % CH_3CN-0.1% TFA (30 min). After lyophilization, the compound **10** was obtained as a yellow oil (4.8 mg, 92%). ^{1}H NMR (400 MHz, CD_3OD): δ 8.58 (s, 1H), 8.13 (s, 1H), 7.84 (d, *J* = 8.0 Hz, 2H), 7.72 (d, *J* = 8.0 Hz, 2H), 7.20 (s, 1H), 3.73 (s, 3H), 3.63–3.57 (m, 11H), 3.48 (m, 2H), 2.93 (t, *J* = 6.0 Hz, 2H), 1.43–1.32 (m, 15H). ^{13}C NMR (150 MHz, $CDCl_3$): δ170.84, 170.21, 165.21, 162.53, 158.63, 154.16, 148.91, 141.87, 139.66, 133.81, 129.56, 120.32, 118.83, 115.95, 102.30, 80.08, 71.53, 71.32, 70.39, 70.10, 54.50, 42.66, 40.88, 40.52, 38.74, 32.33, 30.57, 28.80, 23.08. HR-ESI-MS: calcd. For $C_{33}H_{45}N_9O_6S$ $[M+H]^+$ = 696.3286, obsd. = 696.3281.

Compound **1**: To a solution of compound **10** (0.4 mg, 0.5 μmol) in dry DMF (50 μL), Alexa Fluor™ 647 NHS ester (1.0 mg, 0.8 μmol, 1.6 eq.) and DIPEA (1 μL, 5.7 μmol, 11 eq.) were added and the reaction mixture was stirred at RT for 1 h. The reaction mixture was purified by reversed-phase HPLC with a linear gradient of 10-40 % CH_3CN-0.1% TFA (30 min). After lyophilization, the residue was dissolved in CH_2Cl_2/TFA (1/1) (1 mL) and stirred at RT for 30 min. After azeotropic removal of TFA with toluene (2 mL x 2), the solvent was removed to afford **1** as a blue oil (0.32 mg, 44%). HR-ESI-MS: calcd. for $C_{64}H_{81}N_{11}O_{17}S_5$ $[M-3H+2Na]^-$ = 1478.3982, obsd. = 1478.4004. calcd. for $[M-2H+Na]^-$ = 1456.4162, obsd. = 1456.4191.

Synthesis of 2

Methyl (*S*)-4-(2-(((benzyloxy)carbonyl)amino)-6-((*tert*-butoxycarbonyl)amino)hexanamido)butanoate (**11**): To a solution of *N*-*α*-benzyloxycarbonyl-*N*-*ε*-*tert*-butoxycarbonyl-*L*-lysine (500 mg, 1.31 μmol, 1.0 eq.) in dry DMF (13 mL) were added 4-aminobutyric acid methyl ester hydrochloride (240 mg, 1.58 μmol, 1.2 eq.), 1-(3-dimethylaminopropyl)-3-ethylcarbodiimide hydrochloride (EDC·HCl: 328 mg, 1.71 μmol, 1.3 eq.), 1-hydroxybenzotriazole monohydrate (HOBt·H_2O: 262 mg, 1.71 μmol, 1.3 eq.), and DIPEA (916 μL, 5.26 μmol, 4.0 eq.). After stirring for 10 h, the reaction mixture was diluted with EtOAc (60 mL) and then washed with 0.01 M HCl aq. (20 mL x2), saturated $NaHCO_3$ aq. (20 mL x2) and brine (20 mL). The organic layer was dried over Na_2SO_4, and evaporated under reduced pressure. The residue was purified by silica gel column chromatography ($CHCl_3$:MeOH:AcOH = 100:2:0.5 to 100:5:0.5) to yield compound **11** (613 mg, 97%) as a white solid. ^{1}H NMR (400 MHz, CD_3OD): δ 7.36–7.26 (m, 5H), 5.07 (s, 2H),

4.02–3.98 (m, 5H), 3.64 (s, 3H), 3.31–3.29 (m, 2H), 3.03–2.98 (m, 2H), 2.36–2.32 (m, 2H), 1.81–1.72 (m, 2H), 1.69–1.59 (m, 2H), 1.41 (s, 9H), 1.37–1.31, (m, 4H). ^{13}C NMR (100 MHz, CD_3OD): δ 175.28, 175.03, 158.52, 158.38, 138.13, 129.46, 129.01, 128.90, 128.78, 79.82, 67.67, 56.54, 52.07, 40.95, 39.53, 32.93, 31.91, 30.53, 28.79, 25.64, 24.16.

(*S*)-4-(2-(((Benzyloxy)carbonyl)amino)-6-((*tert*-butoxycarbonyl)amino)hexanamido)butanoic acid **(12):** To a solution of **11** (599 mg, 1.25 mmol) in THF (12.5 mL) was added portionwise 1 M NaOH (1.25 mL, 1.25 mmol, 1.0 eq.) every 1 h for 4 h. After stirring for another 1 h, the pH of reaction mixture was adjusted to pH 3 using 1 M HCl aq. The crude product was extracted with EtOAc (60 mLx1 and 30 mLx2). The organic layer was washed with water (20 mL x 2) and brine (20 mL x 1). The organic layer was dried over Na_2SO_4 and evaporated under reduced pressure. The residue was purified by silica gel column chromatography ($CHCl_3$:MeOH:AcOH = 100:2:0.5) to afford **12** (393 mg, 68%) as a white solid. ^{1}H NMR (400 MHz, CD_3OD) δ 7.34–7.27 (m, 5H), 5.07 (s, 2H), 4.04–4.01 (m, 1H), 3.21 (t, *J* = 6.4 Hz, 2H), 3.00 (t, *J* = 6.4 Hz, 2H), 2.33–2.29 (m, 2H), 1.79–1.62 (m, 4H), 1.41 (s, 9H), 1.36–1.32 (m, 4H). ^{13}C NMR (100 MHz, CD_3OD): δ 176.84, 175.16, 175.01, 158.51, 158.38, 138.09, 129.91, 129.45, 129.20, 129.01, 126.29, 79.83, 67.69, 56.61, 40.94, 39.66, 32.93, 32.07, 30.51, 28.78, 25.67, 24.14.

Ethyl (*S*)-2-(7-(3-(9-(((benzyloxy)carbonyl)amino)-17,17-dimethyl-3,8,15-trioxo-16-oxa-2,7,14-triazaoctadecyl)-1*H*-pyrrol-1-yl)-2,3-dioxo-6-(trifluoromethyl)-3,4-dihydroquinoxalin-1(2*H*)-yl)acetate (**14**): The compound **13** was synthesized according to the literature.[S2,S3] To a solution of **13** (30 mg, 67 μmol, 1.0 eq.) in dry DMF (2.0 mL) were added **12** (40.3 mg, 87 μmol, 1.3 eq.), 1-(3-dimethylaminopropyl)-3-ethylcarbodiimide hydrochloride (EDC·HCl: 16.7 mg, 87 μmol, 1.3 eq.), 1-hydroxybenzotriazole monohydrate (HOBt·H_2O: 13,4 mg, 87 μmol, 1.3 eq.), and DIPEA (47 μL, 269 μmol, 4.0 eq.) under N_2 atmosphere. After stirring for 17 h, the resulting mixture was evaporated under reduced pressure. The resulting residue was dissolved in $CHCl_3$ (100 mL) and washed with 0.01 M HCl aq. (20 mL x 2), saturated $NaHCO_3$ (20 mL x 2) and brine (20 mL). The organic layer was dried over Na_2SO_4 and evaporated under reduced pressure. The residue was purified by silica gel column chromatography ($CHCl_3$:MeOH = 100:5 to 100:10) to afford **14** as a white solid (47.6 mg, 91%). ^{1}H NMR (400 MHz, CD_3OD) δ 7.59 (s, 1H), 7.33–7.19 (m, 6H), 6.81–6.70 (m, 2H), 6.21–6.19 (m, 1H), 5.05–4.95 (m, 4H), 4.24 (s, 2H), 4.20 (q, *J* = 7.2 Hz, 2H), 4.02–3.99 (m, 1H), 3.25–3.18 (m, 2H), 3.03–2.95 (m, 2H), 2.20 (t, *J* =7.2 Hz, 2H), 1.81–1.55 (m, 4H), 1.43 (s, 9H), 1.36–1.28 (m, 4H), 1.25 (t, *J* = 7.2 Hz, 3H). ^{13}C NMR (100 MHz,

CD_3OD): δ 174.70, 174.62, 175.01, 168.22, 158.12, , 157.97, 156.79, 154.62, 137.43, 135.91, 130.49, 129.17, 128.80, 128.60, 125.92, 125.07, 124.81, 123.19, 123.03, 122.87, 122.57, 122.36, 117.08, 115.72, 115.67, 110.29, 79.69, 67.53, 63.08, 58.11, 56.33, 45.37, 40.65, 39.42, 33.98, 32.62, 30.19, 28.75, 26.40, 23.79, 18.25, 14.38.

(*S*)-2-(7-(3-(9-amino-17,17-dimethyl-3,8,15-trioxo-16-oxa-2,7,14-triazaoctadecyl)-1*H*-pyrrol-1-yl)-2,3-dioxo-6-(trifluoromethyl)-3,4-dihydroquinoxalin-1(2*H*)-yl)acetic acid (**15**): To a solution of **14** (44.4 mg, 51.8 μmol) in dry MeOH (2.0 mL) was added 10% Pd/C (20 mg) under N_2 atmosphere. After stirring for 4 h under H_2 atmosphere, the resulting mixture was filtered with a Celite® pad and evaporated under reduced pressure to afford the Cbz deprotected product. The Cbz deprotected product was dissolved in THF (2 mL) and 0.5 M LiOH aq. (0.31 mL, 155 μmol, 3.0 eq.) was added. After stirring for 2 h, the resulting mixture was neutralized with 1 M HCl aq. and evaporated under reduced pressure. The crude product was dissolved in $CHCl_3$: MeOH = 3 :1 (2 mL), then filtered. The filtrate was evaporated under reduced and the residue was purified by silica gel column chromatography ($CHCl_3$:MeOH:28% NH3 aq. = 100:50:3 to 100:100:4) to afford **15** as a white solid (23.7 mg, 66%). ^1H NMR (400 MHz, CD_3OD) δ 7.57 (s, 1H), 7.14 (s, 1H), 6.80 (bs, 1H), 6.76 (bs, 1H), 6.21–6.17 (m, 1H), 4.76 (s, 2H), 4.27, (d, *J* = 14.8 Hz, 1H), 4.22 (d, *J* = 14.8 Hz, 1H), 3.75 (t, *J* = 6.8 Hz, 1H), 3.26–3.17 (m, 2H), 3.02 (t, *J* = 7.2 Hz, 2H), 2.25 (t, *J* = 7.2 Hz, 2H), 1.88–1.69 (m, 4H), 1.53–1.32 (m, 4H), 1.41 (s, 9H). ^{13}C NMR (100 MHz, CD_3OD): δ 174.90, 173.23, 171.20, 158.59, 157.55, 135.93, 131.65, 125.09, 123.53, 122.89, 117.75, 110.49, 79.94, 58.33, 54.68, 40.89, 39.96, 37.18, 34.34, 32.73, 30.54, 28.80, 27.22, 23.34, 18.38.

Compound **2**: To a solution of **15** (1.0 mg, 1.46 μmol, 1.4 eq.) in dry DMF (0.1 mL) was added Alexa Fluor™ 647 NHS ester (1.0 mg, 1.04 μmol, 1.0 eq.) and DIPEA (1.09 μL, 3.13 μmol, 6.0 eq.) under N_2 atmosphere. After stirring for 4 h, the resulting mixture was evaporated under reduced pressure. The crude product was dissolved in CH_2Cl_2 (1.0 mL). To this solution was added TFA (200 μL). After stirring for 1 h, the solvent was removed azeotropically with toluene (200 μL, x3). The crude product was purified by RP-HPLC (YMC-Pack ODS-A, CH_3CN : CH_3COONH_4 = 15 : 85 → 30 : 70, linear gradient over 45 min) to afford **2**. HR-ESI-MS: calcd. for $C_{76}H_{104}FN_9O_{20}S_4$ $[M-2H]^{2-}$ = 716.6977, obsd. = 716.6962.

Synthesis of 3

tert-butyl (15-(8-(4-(4-fluorophenyl)-4-oxobutyl)-4-oxo-1-phenyl-1,3,8-triazaspiro[4.5]decan-3-yl)-12-oxo-3,6,9-trioxa-13-azapentadecyl)carbamate (**17**): The compound **16** was synthesized according to the literature.[S4] To a solution of **16** (122 mg, 0.23 mmol) in CH_2Cl_2 (4 mL) were added TFA (1 mL). After stirring for 1 h at RT under Ar atmosphere, the solvent was removed azeotropically with toluene (1 mL x 3) and $CHCl_3$ (1 mL x 3) to afford the Boc deprotected product. The Boc deprotected product (80 mg, 0.18 mmol, 1 eq.) was dissolved in dry DMF (10 mL), and Boc- 3-(2-(2-(2-aminoethoxy)ethoxy)ethoxy)propanoic acid (76.2 mg, 0.25 mmol, 1.4 eq.), EDC HCl (52.5 mg, 0.27 mol, 1.5 eq.), HOBt H_2O (41.9 mg, 0.25 mmol, 1.5 eq) and DIPEA (283 μL, 1.8 mmol, 10 eq.) were added. After stirring at RT for 16 h, Et_2O (40 mL) was added and the organic layer was washed with sat. $NaHCO_3$ aq. (15 mL x 3). The organic layer was dried over $MgSO_4$ and evaporated under reduced pressure to afford compound **17** (72 mg, 0.095 mmol, 53%) as a white solid. ^{1}H NMR (400 MHz, $CDCl_3$) δ 8.02 (dd, *J* = 7.2 Hz, 5.6 Hz, 2H), 7.24 (t, *J* = 8.4 Hz, 2H), 7.13 (t, *J* = 8.4 Hz, 2H), 6.90 – 6.85 (m, 3H), 5.16 (br, 1H), 4.74 (s, 2H), 3.71 (t, *J* = 6.0 Hz, 2H), 3.64 – 3.50 (m, 15 H), 3.31 – 3.20 (m, 2H), 3.01 (t, *J* = 7.2 Hz, 2H), 2.81 – 2.79 (m, 4H), 2.60 – 2.44 (m, 6H), 1.96 (t, *J* = 7.2 Hz, 2H), 1.44 (s, 9H), 0.89 (t, *J* = 6.4 Hz, 2H)

3-(2-(2-(2-aminoethoxy)ethoxy)ethoxy)-*N*-(2-(8-(4-(4-fluorophenyl)-4-oxobutyl)-4-oxo-1-phenyl-1,3,8-triazaspiro[4.5]decan-3-yl)ethyl)propanamide (**18**): To a solution of **17** (72 mg, 95 μmol) in CH_2Cl_2 (9 mL) were added TFA (1 mL). After stirring for 1 h at RT under Ar atmosphere, the solvent was removed azeotropically with toluene (6 mL x 1) and CH_2Cl_2 (3 mL x 3). The residue was purified by RP-HPLC (Cosmosil 5C18AR2, CH_3CN-0.1% TFA : H_2O-0.1% TFA = 20 : 80 → 70 : 30, linear gradient over 50 min) to afford **18** (8.0 mg, 9% as 3TFA salts) as a yellow oil. ^{1}H NMR (400 MHz, CD_3OD) δ 8.08 (dd, *J* = 8.8 Hz, 5.6 Hz, 2H), 7.30 (t, *J* = 8.4 Hz, 2H), 7.23 (t, *J* = 8.4 Hz, 2H), 7.02 (d, J = 8.8 Hz, 2H), 6.93 (t, *J* = 5.6 Hz, 1H), 4.84 (s, 2H), 3.82 (ddd, *J* =13.2 Hz, 2.8 Hz, 2H) 3.70 – 3.48 (m, 18H), 3.40 (t , *J* = 5.6 Hz, 2H), 3.26 – 3.19 (m, 4H), 3.01 (t, 4.8 Hz, 2H), 2.79 (br, 2H),2.44 (t, *J* = 6.0 Hz, 2H), 2.16(br, 2H), 2.04 (br, 2H)

Compound **3**: Compound **18** (8 mg, 12.5 μmol, 1 eq.) was dissolved in dry DMF (2 mL), and Fmoc-Lys(Mtt)-OH (10.5 mg, 16.8 μmol, 1.35 eq.), EDC HCl (4.2 mg, 21.9 μmol, 1.8 eq.), HOBt H_2O (2.5 mg, 16.3 μmol, 1.31 eq.), and DIPEA (25.7 μL, 165.7 μmol, 13.3 eq.) were added. After stirring at RT for 18 h, CH_2Cl_2 (10 mL) was added and the organic layer was washed with sat. $NaHCO_3$ aq. (2 mL x 2). The organic layer was dried over Na_2SO_4 and evaporated under reduced pressure. The residue was dissolved with dry DMF (0.8 mL) and piperidine (0.2 mL). The mixture

was stirred at RT for 90 min and the solvent was removed by evaporation under reduced pressure to give a Fmoc deprotected product. The Fmoc deprotected product (1.7 mg, 1.66 μmol, 1 eq.) was dissolved in dry DMF (1 mL) and Alexa Fluor™ 647 (1.9 mg, 2.21 μmol, 1.33 eq.), EDC HCl (0.49 mg, 2.56 μmol, 1.5 eq.), HOBt H_2O (0.38 mg, 2.48 μmol, 1.5 eq.) and DIPEA (6 μL, 38.7 μmol, 23.4 eq.) were added under Ar atmosphere. After stirring at RT for 15 h, the solvent was removed by evaporation under reduced pressure. The crude product was dissolved in CH_2Cl_2 (0.54 mL) and HFIP (0.13 mL) was added. After stirring for 1 h, the solvent was removed and the residue was purified by RP-HPLC (Cosmosil 5C18AR2, CH_3CN-0.1% TFA : H_2O-0.1% TFA = 5 : 95 → 55 : 45, linear gradient over 50 min) to afford **3** as a blue film. HR-ESI-MS: calcd. for $C_{76}H_{104}FN_9O_{20}S_4$ $[M-3H+Na]^{2-}$ = 803.8049, obsd. =803.8060.

Synthesis of 4

tert-butyl (2-(2-(2-(2-((6-(2-(4-cyano-*N*-methylbenzamido)thiazol-4-yl)pyrimidin-4-yl)amino)ethoxy)ethoxy)ethoxy)ethyl)carbamate (**19**): To a solution of **8** (7.5 mg, 21 μmol) in DMF (0.1 mL) was added *tert*-butyl (2-(2-(2-(2-aminoethoxy)ethoxy)ethoxy)ethyl)carbamate (31 mg, 105 μmol, 5.0 eq.) and K_2CO_3 (15.0 mg, 105 μmol, 5.0 eq.). The resulting mixture was stirred at 50 °C. After completion of the reaction (22.5 h), DMF was removed in vacuo. The resulting residue was purified with flash column chromatography (CH_2Cl_2:MeOH = 9:1) to afford the compound **19** as yellow oil (4.5 mg, 35%). ^1H NMR (600 MHz, $CDCl_3$): δ 8.58 (s, 1H), 7.96 (s, 1H), 7.83 (d, *J* = 4.0 Hz, 2H), 7.70 (d, *J* = 4.0 Hz, 2H), 7.11 (s, 1H), 3.73–3.63 (m, 15H), 3.55 (t, *J* = 5.2 Hz, 2H), 3.31 (m, 2H), 1.42 (s, 9H). ^{13}C NMR (150 MHz, $CDCl_3$): δ 168.43, 163.34, 159.89, 158.61, 157.08, 156.05, 148.34, 138.71, 132.61, 128.23, 117.71, 116.25, 114.90, 100.44, 77.03, 70.63-69.69, 40.99, 40.52, 38.12, 28.47. HR-ESI-MS: calcd. for $C_{29}H_{37}N7O_6S$ $[M+Na]^+$ = 634.2418, obsd. = 634.2410.

Compound **4**: To a solution of compound **19** (0.4 mg, 0.65 μmol, 1.0 eq.) in CH_2Cl_2 (0.5 mL), TFA (0.5 mL) was added and the reaction mixture was stirred at RT for 30 min. After azeotropic removal of TFA with toluene (2 mL x 2), the residue was dissolved in dry DMF (150 μL). To the solution, Alexa Fluor™ 647 NHS ester (1.0 mg, 0.8 μmol, 1.2 eq.) and DIPEA (1 μL, 6.3 μmol, 10 eq.) were added and the reaction mixture was stirred at RT for 1 h. The reaction mixture was purified by reversed-phase HPLC with a linear gradient of 20-40 % CH_3CN-0.1% TFA (20 min) to afford **4** as blue oil (0.2 mg, 24%). HR-ESI-MS: calcd. for $C_{60}H_{73}N_9O_{17}S_5$ $[M-H]^-$ = 1350.3655, obsd. = 1350.3677.

Synthesis of 5

Compound **5:** To a solution of Alexa Fluor 647 (0.5 mg, 0.5 μmol) in dry DMF (50 μL), *N,N,N′,N′*-Tetramethyl-*O*-(*N*-succinimidyl)uronium tetrafluoroborate (TSTU: 0.9 mg, 2.9 μmol, 6 eq.) and triethylamine (3.2 μL, 23 μmol, 40 eq.) were added. After stirring at RT for 1 h, propane-1,3-diamine hydrochloride (0.9 mg, 5.8 μmol, 10 eq.) was added. After stirring at RT for 30 min, the reaction mixture was diluted with water and purified by reversed-phase HPLC with a linear gradient of 10-40 % CH_3CN-0.1% TFA (30 min) to afford **5**. HR-ESI-MS: calcd. for $C_{39}H_{54}N_4O_{13}S_4$ $[M-3H+Na]^{2-}$ = 467.1122, obsd. = 467.1123.

Synthesis of 6

tert-butyl (2-(2-(2-((6-(2-(4-(3-fluoropropoxy)-*N*-methylbenzamido)thiazol-4-yl)pyrimidin-4-yl)amino)ethoxy)ethoxy)ethyl)carbamate (**21**): The compound **20** was synthesized according to the literature.[S5] To a solution of compound **20** (108 mg, 0.265 mmol) in DMF (1 mL), *tert*-butyl (2-(2-(2-aminoethoxy)ethoxy)ethyl)carbamate (85 mg, 0.345 mmol, 1.3 eq.) and K_2CO_3 (55 mg, 0.398 mmol, 1.5 eq.) were added. After stirring at 60 °C overnight under Ar atmosphere, CH_2Cl_2 (50 mL) was added and the organic layer was washed with brine (25 mL x2). The organic layer was dried over Na_2SO_4 and the solvent was evaporated. The residue was purified by silica gel column chromatography (CH_2Cl_2 : CH3OH = 96 : 4) to yield compound **21** as a white solid (132 mg, 0.214 mmol, 81%). ^{1}H NMR (400 MHz, $CDCl_3$): δ 8.61 (d, *J* = 1.1 Hz, 1H), 7.93 (s, 1H), 7.60 (d, *J* = 8.8 Hz, 2H), 7.14 (s, 1H), 7.02 (d, *J* = 8.8 Hz, 2H), 4.69 (dt, *J* = 47.0, 5.7 Hz 2H), 4.20 (t, *J* = 6.1 Hz, 2H), 3.80 (s, 3H), 3.77–3.74 (m, 2H), 3.69–3.66 (m, 6H), 3.39 (t, *J* = 5.1 Hz, 2H), 3.38–3.37 (m, 2H), 2.32–2.15 (m, 2H), 1.46 (s, 9H). ^{13}C NMR (150 MHz, $CDCl_3$, 60 °C): δ 170.04, 163.28, 160.98, 160.80, 158.39, 157.40, 155.98, 148.06, 129.81, 126.65, 115.58, 114.44, 99.91, 80.31, 79.09, 70.36, 70.29, 70.23, 69.58, 63.92, 40.96, 40.56, 38.42, 30.27, 28.33.

tert-butyl (***S***)-(5-amino-6-((2-(2-(2-((6-(2-(4-(3-fluoropropoxy)-*N*-methylbenzamido)thiazol-4-yl)pyrimidin-4-yl)amino)ethoxy)ethoxy)ethyl)amino)-6-oxohexyl)carbamate (**22**): To a solution of compound **21** (40 mg, 65 μmol) in CH_2Cl_2 (0.5 mL), TFA (0.5 mL) was added and the reaction solution was stirred at RT for 30 min. After azeotropic removal of TFA with toluene (2 mL x 2), the residue was dissolved in dry DMF (0.65 mL). Then, Fmoc-Lys(Boc)-OH (45 mg, 97 μmol, 1.5 eq.), HBTU (37 mg, 97 μmol, 1.5 eq.) and DIPEA (33 μL, 190 μmol, 2.9 eq.) were added. After stirring the reaction mixture at RT overnight, piperidine (98 μL) was added and the reaction mixture was

stirred at RT for another 2 h. The solvent was evaporated and the residue was dissolved in CH_2Cl_2 (30 mL). The organic layer was washed with sat. $NaHCO_3$ aq. (15 mL x 2) and brine (15 mL x2). After drying over Na_2SO_4, the solvent was evaporated and the residue was purified by preparative TLC (CH_2Cl_2 : MeOH : NH3 aq. = 150 : 10 : 1.6) to yield compound **22** as a pale yellow form (46 mg, 60 μmol, 93%). ^{1}H NMR (400 MHz, $CDCl_3$): δ 855 (d, *J* = 1.1 Hz, 1H), 7.90 (s, 1H), 7.57 (d, *J* = 8.8 Hz, 2H), 7.09 (d, *J* = 1.5 Hz, 1H), 6.99 (d, *J* = 8.8 Hz, 2H), 4.66 (dt, *J* = 47.0, 5.7 Hz 2H), 4.17 (t, *J* = 6.1 Hz, 2H), 3.76 (s, 3H), 3.74–3.70 (m, 2H), 3.69–3.53 (m, 8H), 3.50–3.44 (m, 4H), 3.35–3.32 (m, 1H), 3.11–3.06 (m, 2H), 2.29–2.12 (m, 2H), 1.57–1.29 (m, 15H). ^{13}C NMR (150 MHz, $CDCl_3$, 60 °C): δ 175.14, 170.29, 163.51, 161.22, 161.14, 158.59, 157.77, 156.1, 148.20, 130.05, 126.89, 115.98, 114.67, 100.01, 80.51, 79.18, 70.56, 70.39, 70.14, 69.72, 64.13, 55.53, 41.20, 40.57, 39.05, 38.65, 34,96, 30.51, 30.04, 28.55, 23.01.

Compound **6**: To a solution of compound **22** (3.0 mg, 4.0 μmol, 5 eq.) in dry DMSO (120 μL), Alexa Fluor™ 647 NHS ester (1.0 mg, 0.8 μmol, 1.0 eq.) and DIPEA (1 μL, 5.7 μmol, 7.2 eq.) were added. After stirring at RT overnight, the solvent was removed by lyophilization. The residue was dissolved in TFA/CH_2Cl_2 (1/1) (1 mL) and stirred at RT for 30 min. The solvent and TFA was removed by evaporation and the residue was purified by reversed-phase HPLC with a linear gradient of 15-30 % CH_3CN-0.1% TFA (20 min) to afford **6** as a blue film. HR-ESI-MS: calcd. for $C_{66}H_{87}FN_{10}O_{18}S_5$ $[M-3H+2Na]^-$ = 1529.4353, obsd. = 1529.4491. calcd. for $[M-3H+Na]^{2-}$ = 753.2231, obsd. =753.2218.

Synthesis of S1 and S2

To a solution of **S6** (3.0 mg, 1.96 μmol) in CH_2Cl_2 (0.4 mL) were added TFA (0.1 mL). After stirring for 1 h at RT, the solvent was removed azeotropically with CH_3CN:toluene = 1:1 (400 μL x 3) to afford the Boc deprotected product. This Boc deprotected product was used directly for the next step without further purification.

Compound **S1**: To a solution of the half amount of Boc deprotected product (0.98 μmol, 1.0 eq.) in dry DMF (0.2 mL), **S7**[S6] (1.20 mg, 3.00 μmol) and DIPEA (5.1 μL, 29.3 μmol) were added and the reaction mixture was stirred at RT under Ar atmosphere. After stirring for 17 h, the reaction mixture was purified by reversed-phase HPLC with a linear gradient of 20-70 % CH_3CN (50 min) to afford **S1** as blue powder (0.430 μmol, 41%, determined by the measurement of UV-absorbance). HR-ESI-MS: calcd. for $C_{71}H_{85}B_1F_1N_{10}O_{21}S_5$ $[M-3H]^{3-}$ = 534.1541, obsd. = 534.1541.

Compound **S2**: To a solution of benzoic acid (0.60 mg, 4.91 μmol), COMU (2.1 mg, 4.90 μmol), and DIPEA (1.3 μL, 7.46 μmol) in dry DMF 200 μL, the half amount of Boc deprotected product (0.98 μmol, 1.0 eq.) in dry DMF (0.2 mL) was added and the reaction mixture was stirred at RT for 2 h. To this reaction mixture, additional activated mixture containing benzoic acid (1.8 mg, 14.7 μmol), COMU (3.1 mg, 7.24 μmol), and DIPEA (1.3 μL, 7.46 μmol) in dry DMF 100 μL was added. After stirring for 2 h, the reaction mixture was purified by reversed-phase HPLC with a linear gradient of 20-70 % CH_3CN (50 min) to afford **S2** as blue powder (0.702 μmol, 72% determined by the measurement of UV-absorbance). HR-ESI-MS: calcd. for $C_{70}H_{82}F_1N_{10}O_{18}S_5$ $[M-3H]^{3-}$ = 509.8138, obsd. = 509.8133.

Synthesis of S3

To a solution of compound **10** (0.5 mg, 0.6 μmol) in dry DMF (50 μL), Alexa Fluor™ 555 NHS ester (1.0 mg, 0.8 μmol, 1.3 eq.) and DIPEA (2 μL, 13 μmol, 22 eq.) were added and the reaction mixture was stirred at RT overnight. The reaction mixture was purified by reversed-phase HPLC with a linear gradient of 20-50 % CH_3CN-0.1% TFA (30 min). After lyophilization, a half of the residue was dissolved in CH_2Cl_2/TFA (1/1) (1 mL) and stirred at RT for 50 min. The reaction mixture was purified by reversed-phase HPLC with a linear gradient of 20-50 % CH_3CN-0.1% TFA (30 min) to afford **S3** as a magenta oil (0.02 mg, 4.5%). HR-ESI-MS: calcd. for $C_{62}H_{78}N_{11}O_{17}S_5$ $[M-H]^-$ = 1408.4186, obsd. = 1408.4186.

Synthesis of S4

S9: The compound **S8** was synthesized according to the literature.[S7] To a stirred solution of compound **S8** (9.4 mg, 16.5 μmol) in CH_2Cl_2 (400 μL), TFA (100 μL) was added and the reaction solution was stirred at RT for 1.5 h. After azeotropic removal of TFA with CH_3CN:toluene = 1:1 (400 μL x 3) to afford the Boc deprotected product, the residue was dissolved in dry DMF (0.2 mL) and DIPEA (86 μL, 494 μmol). Additional activated mixture containing Fmoc-Lys(Mtt)-OH (12.4 mg, 19.8 μmol), EDC-HCl (4.6 mg, 23.8 μmol), HOBt-H_2O (3.6 mg, 23.8 μmol), and DIPEA (14.4 μL, 82.7 μmol) in dry DMF 300 μL was stirred at RT for 2.5 h and then this activated mixture was added to the mixture of the Boc deprotected product. After stirring at RT under Ar atmosphere for 1.5 h, the solvent was evaporated. The residue was dissolved in EtOAc (30 mL) and the obtained organic phase was washed with saturated $NaHCO_3$ aq. (5 mL) and brine (5 mL). The organic layer was dried over Na_2SO_4, and evaporated under reduced pressure. After dissolving

the residue in DMF (400 µL), piperidine (100 µL) was added and the mixture was stirred at RT under Ar atmosphere for 0.5 h. The solvent was evaporated and the residue was purified by reversed-phase HPLC with a linear gradient of 30-100 % CH_3CN (55 min). After lyophilization, the compound **S10** was obtained as a white solid (5.2 mg, 6.08 µmol, 37%). ^{1}H NMR (600 MHz, DMSO-d_6): δ 8.11 (s, 1H), 7.97 (t, *J* = 6.0 Hz, 1H), 7.46 (s, 1H), 7.42–7.32 (m, 4H), 7.25–7.22 (m, 6H), 7.12 (t, *J* = 7.2 Hz, 2H), 7,04 (d, *J* = 8.4 Hz, 2H), 6.81 (s, 1H), 6.05 (s, 2H), 4.12 (t, *J* = 7.2 Hz, 2H), 3.07–3.01 (m, 3H), 2.22 (s, 3H), 1.91 (d, *J* = 6 Hz, 2H), 1.82 (quin, *J* = 7.2 Hz, 2H), 1.49–1.40 (m, 4H), 1.29–1.24 (m, 2H). ^{13}C NMR (150 MHz, DMSO-d_6): δ175.05, 155.20, 152.94, 150.94, 148.83, 148.50, 146.43, 143.99, 143.28, 134.91, 128.31, 128.22, 127.58, 125.87, 119.51, 118.65, 111.25, 102.43, 91.09, 70.09, 54.78, 43.36, 41.17, 35.75, 35.17, 30.11, 29.63, 23.33, 20.49. HR-ESI-MS: calcd. For $C_{41}H_{44}I_1N_8O_3S_1$ $[M+H]^+$ = 855.2296, obsd. = 855.2300.

Compound **S4**: To a solution of **S9** (1.2 mg, 1.40 µmol) and BODIPY FL NHS (0.75 mg, 1.40 µmol) in dry DMF (0.4 mL) was added DIPEA (2.4 µL, 13.8 µmol) under Ar atmosphere. After stirring for 17 h, the solvent was removed azeotropically with CH_3CN:toluene = 1:1 (400 µL x 3). The residue was dissolved in CH_2Cl_2 (0.3 mL) and hexafluoroisopropanol (HFIPA, 0.3 mL). The mixture was stirred at RT for 2.5 h and then TFA (5 µL, 65.3 µmol) was added to the reaction mixture. After stirring at RT for 2 h, TEA (27 µL, 194 µmol) was added to the reaction mixture for neutralization and the solvent was removed under reduced pressure. The resulting residue was purified by reversed-phase HPLC with a linear gradient of 25-70 % CH_3CN (45 min) to afford **S1** as orange powder (0.118 µmol, 8.4%, determined by the measurement of UV-absorbance). HR-ESI-MS: calcd. for $C_{35}H_{41}B_1F_2I_1N_{10}O_4S_1$ $[M+H]^+$ = 872.2170, obsd. = 872.2181.

Synthesis of S5

Compound **S5**: To a stirred solution of compound **S8** (10.7 mg, 18.8 µmol) in CH_2Cl_2 (400 µL), TFA (100 µL) was added and the reaction solution was stirred at RT for 1.5 h. After azeotropic removal of TFA with CH_3CN:toluene = 1:1 (400 µL x 3) to afford the Boc deprotected product, the residue was dissolved in dry DMF (0.2 mL) and DIPEA (86 µL, 494 µmol). Additional activated mixture containing Fmoc-Gly-OH (8.4 mg, 19.8 µmol), COMU (12.0 mg, 28.1 µmol), and DIPEA (9.8 µL, 56.3 µmol) in dry DMF 200 µL was stirred at RT for 1.5 h and then this activated mixture was added to the mixture of the Boc deprotected product. After stirring at RT under Ar atmosphere for 1.5 h, the solvent was evaporated. The residue was dissolved in EtOAc (20 mL) and the obtained organic phase was washed with saturated $NaHCO_3$ aq. (5 mL) and brine (5 mL). The

organic layer was dried over Na_2SO_4, and evaporated under reduced pressure. After dissolving the residue in DMF (400 μL), piperidine (100 μL) was added and the mixture was stirred at RT under Ar atmosphere for 20 min. The solvent was evaporated under reduced pressure. The residue was dissolved in EtOAc (20 mL) and the obtained organic phase was washed with saturated $NaHCO_3$ aq. (5 mL) and brine (5 mL). The organic layer was dried over Na_2SO_4, and evaporated under reduced pressure. The residue was purified by preparative TLC ($CHCl_3$:MeOH:aq. NH_3 = 9:1:0.1) to afford the Fmoc deprotected product as a white solid (1.6 mg, 3.03 μmol, 16%). The crude products were used directly for the next step without further purification. HR-ESI-MS: calcd. For $C_{17}H_{17}IN_7O_3S_1Na_1$ $[M+H]^+$ = 549.0050, obsd. = 549.0594. To a solution of the Fmoc deprotected product (1.5 mg, 2.84 μmol) and BODIPY FL NHS (1.1 mg, 2.83 μmol) in dry DMSO (0.21 mL) was added DIPEA (4.9 μL, 28.1 μmol) under Ar atmosphere. After stirring for 17 h, the reaction mixture was purified by reversed-phase HPLC with a linear gradient of 25-70 % CH_3CN (45 min) to afford **S5** as orange powder (0.50 μmol, 2.7% for 4 steps, determined by the measurement of UV-absorbance). HR-ESI-MS: calcd. for $C_{31}H_{31}B_1F_2I_1N_9O_4S_1$ $[M+Na]^+$ = 823.1254, obsd. = 823.1253.

Preparation of Nb381-Ax647 (Probe 7):

Preparation of Plasmid Construct

For chemical dye conjugation, tandem GS linker and the Cys residue were introduced in front of the His tag sequence using primers, 5'- GGG TCC TGT CAC CAC CAT CAC CAT CAC GAA CCT GAA GCC -3'/ 5'- GCC ACC GCC GCT GCC GCC ACC CCC GGA -3' and Q5 Site-Directed Mutagenesis Kit (New England Biolabs) to confer pMESy4-PelB-Nb381-$(GS)_2$-Cys-6His (Figure S22). The DNA sequence of the plasmid construct was verified by dye-terminator DNA sequencing (Genewiz).

Protein Expression and Purification

The plasmid pMESy4-PelB-Nb381-$(GS)_2$-Cys-6His was transformed into a BL21(DE3) strain using the standard heat-shock protocol. The transformed BL21(DE3) cells were grown in a Luria-Bertani (LB) medium containing 50 μg/mL kanamycin for 16 h at 37 ºC as a starting culture. The starting culture (1 mL) was transferred into a fresh Terrific broth (TB) medium and incubated at 37 ºC until an OD at 600 nm of ~ 0.8 was reached at which time, cells were induced with 1 mM IPTG. Cells were incubated for another 5 h at 37 °C, pelleted by centrifugation, and frozen at -20 °C overnight. Cells were resuspended in a PBS buffer (Thermo Fisher) on ice bath and lysed

using standard sonication protocols. The soluble fractions were loaded onto TALON beads (Takara Bio) pre-equilibrated with PBS. After washing the column with 10 volumes of PBS, the target protein was eluted with PBS containing 500 mM imidazole. The excess imidazole was removed by dialysis using 3.5 K membrane filter (Spectrum Laboratories Inc.). The purified protein was concentrated using 10 K Amicon centrifuge filter (Millipore). Prior to protein-dye conjugation, purified Nb381-$(GS)_2$-Cys recombinant protein was treated using endotoxin removal spin column (Thermo Fisher). The purity and MW of protein were confirmed by SDS-PAGE analysis.

Alexa Fluor 647 maleimide conjugation

To a solution of Nb381-$(GS)_2$-Cys in PBS (300 μM, 500 μL) containing 10 mM TCEP, Alexa Fluor 647 maleimide (100 μM) was added and the reaction mixture was incubated at 24 °C overnight under the dark. The nanobody-dye conjugate was purified using a TOYOPEARL HW-40F size-exclusion column (TOSOH) and Hi-Trap QHP anion exchange column (GE Healthcare). The purity and MW of nanobody-dye conjugate was confirmed by SDS-PAGE, in Gel fluorescence and MALDI-TOF MS analysis (Figures S23 and 24).

Expression of receptors in HEK293T cells.

HEK293T cells (ATCC) were cultured in Dulbecco's modified Eagle's medium (DMEM) supplemented with 10% fetal bovine serum (Sigma Aldrich), penicillin (100 units mL^{-1}), streptomycin (100 μg mL^{-1}) and amphotericin B (250 ng mL^{-1}) and incubated in a 5% CO_2 humidified chamber at 37 °C. For expression of each receptor, HEK293T cells (2.0×10^5 cells) plated on a 3.5-cm dish (Corning) were transfected with each expression vector for HA-tag fused mGlu1[S8] using Lipofectamine 2000 (Invitrogen) according to the manufacturer's instructions.

Confocal imaging of HEK293T cells with *FixEL* probe.

After 24 h of transfection, the cells were dissociated by treating with TrypLE Express (Gibco) and re-seeded on 35 mm glass-bottom dishes (Iwaki) pretreated with poly-L-lysine. After 24 h of the re-seeding, the cells were washed twice with PBS(–) (Wako) then 100 μL of PBS(–) containing a probe (2 μM) was added into the cell cultured dish. After incubation at RT for 5 min, 100 μL of 4% PFA/PBS(–) was added. After incubation at RT for 30 min, cells were washed with DMEM at 37 °C. After HA-tag staining (DyLight550 anti-HA tag, ab117502), fluorescence imaging of the cells was performed using CLSM equipped with a 63×, NA = 1.40 oil objective and a GaAsP detector

(the 561 nm excitation for DyLight550 and the 633 nm excitation for Alexa Flour 647 derived from a white laser).

Determination of an apparent affinity constant of the probes of mGlu1 ligands.

The HEK293T cells transiently transfected with HA-tag fused mGlu1 on 35 mm glass-bottom dishes were washed twice with PBS(–), and then 150 μL of PBS(–) solutions containing a probe at the different concentrations were added to the dishes. After incubation at RT for 5 min, 150 μL of 4% PFA/PBS(–) was added to each dish. After incubation at RT for 30 min, cells were washed twice with PBS(–). After HA-tag staining, fluorescence imaging was performed. The fluorescence intensity of a probe bound to mGlu1 and HA-staining signal at each concentration was determined by enclosing the regions containing a cell membrane with ROIs and $F = F_{probe}/F_{HA}$ was calculated for each cell (F_{probe} is a fluorescence intensity of probe and F_{HA} is a fluorescence intensity of HA-staining signal) (n = 8 cells in a single dish). After plotting the averaged and background-subtracted value against probe concentrations, the apparent dissociation constant (K_d) and saturation point (F_{sat}) were determined by fitting with the theoretical logistic equation. Finally, the values were normalized by F_{sat}.

$$F = F_{sat} / (1 + (\log [\text{probe}] / \log K_d)^n)$$

$$\log n = h$$

F_{sat} is a saturation point.

F_0 is a fluorescence intensity in the absence of probe.

$F_{background}$ is an averaged fluorescence intensity of background.

h is a Hill's coefficient

Perfusion fixation and brain slices preparation.

Experiments were conducted according to the literature.[S9] Briefly, under the deep anesthesia with isoflurane, mice were perfused transcardially with ice-colded 4% formaldehyde/PBS(–) (pH 7.4) (60 mL). The mouse brain samples were fixed with 4% PFA at 4 °C overnight. After washing with PBS(–) (x3), the brain samples were immersed into 30% sucrose/PBS. The brain slices were prepared using a cryostat (Leica, CM-1950).

For the immunostaining, the brain slices were permeabilized with PBS(–) containing 0.1% triton X-100 for 15 min and blocked with 10% normal goat serum (NGS) in PBS(–) containing 0.1%

triton X-100 for 30 min. Then, primary antibody reaction was conducted with rabbit anti-mGlu1 (Frontier Institute, MSFR104030), rabbit anti-Calbindin (Frontier Institute, CB-28kD), rabbit anti-DRD2 (Frontier Institute, AB_2571596), rabbit anti-5HT2A (ImmunoStar, 24288), rabbit anti-GluD2 (SIGMA, HPA056253), guinea pig anti-Shank2 (Synaptic systems, 162 204) or mouse anti-Synaptophysin (abcam, ab8049) in PBS(–) containing 0.1% triton X-100 at 4 °C overnight. For the immunostaining of GluA2, the brain slice (15 µm thickness) was attached to glass slide and activated with antigen retrieval reagent ImmunoSaver (FUJIFILM Wako) at 80ºC for 20 min. After blocking with 10% NGS in PBS(–) containing 0.1% triton X-100 for 30 min, primary antibody reaction was conducted with mouse anti-GluA2 (Merck, MAB397). Secondary antibody reaction was conducted with a goat anti-guinea pig IgG H&L (Alexa Fluor® 405) (ab175678, for Shank2), goat anti-rmouse IgG H&L (Alexa Fluor® 488) (ab150113, for Synaptophysin), goat anti-rabbit IgG H&L (Alexa Fluor® 488) (ab150077, for mGlu1, calbindin, DRD2, 5HT2A, and GluD2) or goat anti-mouse IgG H&L (Alexa Fluor® 594) (abcam, ab150116, for GluA2) in PBS(–) containing 0.1% triton X-100 at r.t. for 1 h.

A proof-of-principle of stimuli-responsive *FixEL* probe in HEK293T cells.

After 24 h of transfection, the cells were dissociated by treating with TrypLE Express (Gibco) and re-seeded on 35 mm glass-bottom dishes (Iwaki) pretreated with poly-L-lysine. After 24 h of the re-seeding, the cells were washed twice with HBSS(+) (nacalai tesque). Only for the condition without H_2O_2 addition, 150 µL of HBSS(+) containing catalase (from bovine liver, wako; 740 units/mL) was added and incubated at 37 °C for 30 min. After removal of the solution, 100 µL of HBSS(+) containing a probe (2 µM) was added into the cell cultured dish. After incubation at RT for 5 min, 100 µL of HBSS(+) containing H_2O_2 (0, 20, 200, or 2000 µM) was added. After incubation at 37 °C for 30 min, 2 mL of 4% PFA/PBS(–) was added. After incubation at RT for 30 min, cells were washed with DMEM at 37 °C. Fluorescence imaging of the cells was performed using CLSM equipped with a 40×, NA = 1.30 oil objective and a GaAsP detector (the 488 nm excitation for EGFP and the 633 nm excitation for Alexa Flour 647 derived from a white laser).

HPLC analysis of the extract from probe-administered mouse brain.

After probe **1** (mGlu1) was injected into the mouse cerebellum and incubated for 6h, the whole cerebellum was isolated and stored at -80 °C overnight. 1 mL of 80% methanol/H_2O (vol./vol.) was added and homogenized with an ultrasonic ruptor (BRANSON Sonifier Model SFX150HH). After incubation at 37 °C for 15 min and centrifugation (15,000×g), the supernatant was dried with

SpeedVac. RP-HPLC analysis was conducted on Shimadzu Nexera system equipped with a fluorescent detector (640/660 nm (Ex/Em)) with a linear gradient of 0-40% CH_3CN/10 mM NH_4OAc aq. (40 min) and 40-100% (5 min).

Determination of an apparent affinity constant of probe 7 (GluD2).

The HEK293T cells transiently transfected with GluD2 on 35 mm glass-bottom dishes were washed twice with PBS(–), and then PBS(–) solutions containing a probe at appropriate concentrations which yield the final concentrations of 0.1, 0.3, 1, 3, 10, 30, 100, 300, and 1000 nM, were sequentially added to the dishes. For each condition of probe concentrations, fluorescence imaging of cells was performed using CLSM equipped with a 40×, NA = 1.30 oil objective and a GaAsP detector (the 633 nm excitation for Alexa Flour 647 derived from a white laser). The intensity of Ax647 signal at each concentration was determined by enclosing the regions containing a cell membrane with ROIs (n = 10 cells in a single dish). The intensity values were normalized by the intensity of the condition with 1000 nM. The apparent dissociation constant (K_d) and saturation point (F_{sat}) were determined by fitting with the theoretical logistic equation.

Clearing of mouse brain tissue with 3DISCO protocol.

Experiments were conducted according to the literature.[S10] The fixed brain sample with 4% PFA/PBS(–) was treated in the mixture of THF/H_2O (vol./vol.) at a series of concentrations (50, 70, 80 (each for 12 h) and 100% (12 h × 3) with shaking at RT on a turning table under the dark. Finally, the brain sample was immersed in dibenzyl ether for refractive index matching for 1–2 day before CLSM imaging. 3D images were reconstructed from z-stacked images using Leica LAS-X. Compression in the z-direction caused by spherical aberration was corrected according to the following formula.[S11]

$$d'/d = \{\tan (\sin^{-1} (0.5\ NA/\ n_1))\}/\ \{\tan (\sin^{-1} (0.5\ NA/\ n_2))\}$$

d' is the actual focal position.

d is the expected focal position.

n_1 is the refractive index of air (1.00).

n_2 is the refractive index of DBE (1.54).

Confocal imaging of SK-BR-3 cells with *FixEL* probe.

SK-BR-3 cells were seeded on 35 mm glass-bottom dishes (Iwaki) pretreated with poly-L-lysine. The cells were washed twice with PBS(–) then 100 μL of PBS(–) containing a probe (100 nM) was added into the cell cultured dish. After incubation at RT for 10 min, 100 μL of 4% PFA/PBS(–) was added. After incubation at RT for 30 min, cells were incubated in McCoy's 5A (modified) medium (Thermo Fisher Scientific) containing 10% FBS at 37 °C overnight to remove non-fixed probes. Fluorescence imaging of the cells was performed using CLSM equipped with a 63×, NA = 1.40 oil objective and a GaAsP detector (488 nm excitation for BODIPY-FL).

Supplemental References

S1. Fujinaga, M., Yamasaki, T., Maeda, J., Yui, J., Xie, L., Nagai, Y., Nengaki, N., Hatori, A., Kumata, K., Kawamura, K., et al. (2012). Development of N-[4-[6-(isopropylamino)pyrimidin-4-yl]-1,3-thiazol-2-yl]-N-methyl-4-[11C]methylbenzamide for positron emission tomography imaging of metabotropic glutamate 1 receptor in monkey brain. J. Med. Chem. *55*, 11042–11051. https://doi.org/10.1021/jm301597s.

S2. Lubisch, W., Benl, B., and Hofmann, H.P. (1997). Pyrrolylquinoxalinediones: The importance of pyrrolic substitution on AMPA receptor binding. Bioorg. Med. Chem. Lett. *7*, 1101–1106. https://doi.org/10.1016/S0960-894X(97)00170-4.

S3. Lubisch, W., Behl, B., and Hofmann, H.P. (1996). Pyrrolylquinoxalinediones: A new class of AMPA receptor antagonists. Bioorg. Med. Chem. Lett. *6*, 2887–2892. https://doi.org/10.1016/S0960-894X(96)00534-3.

S4. Tomlinson, I.D., Kovtun, O., Crescentini, T.M., and Rosenthal, S.J. (2019). Biotinylated-spiperone ligands for quantum dot labeling of the dopamine D2 receptor in live cell cultures. Bioorg. Med. Chem. Lett. *29*, 959–964. https://doi.org/10.1016/j.bmcl.2019.02.024.

S5. Fujinaga, M., Yamasaki, T., Yui, J., Hatori, A., Xie, L., Kawamura, K., Asagawa, C., Kumata, K., Yoshida, Y., Ogawa, M., et al. (2012). Synthesis and evaluation of novel radioligands for positron emission tomography imaging of metabotropic glutamate receptor subtype 1 (mGluR1) in rodent brain. J. Med. Chem. *55*, 2342–2352. https://doi.org/10.1021/jm201590g.

S6. Li, J., Li, Y., He, Q., Li, Y., Li, H., and Liu, L. (2014). One-pot native chemical ligation of peptide hydrazides enables total synthesis of modified histones. Org. Biomol. Chem. *12*, 5435–5441. https://doi.org/10.1039/c4ob00715h.

S7. Tamura, T., Ueda, T., Goto, T., Tsukidate, T., Shapira, Y., Nishikawa, Y., Fujisawa, A., and Hamachi, I. (2018). Rapid labelling and covalent inhibition of intracellular native proteins using ligand-directed N-acyl-N-alkyl sulfonamide. Nat. Commun. *9*, 1870. https://doi.org/10.1038/s41467-018-04343-0.

S8. Kiyonaka, S., Kubota, R., Michibata, Y., Sakakura, M., Takahashi, H., Numata, T., Inoue, R., Yuzaki, M., and Hamachi, I. (2016). Allosteric activation of membrane-bound glutamate receptors using coordination chemistry within living cells. Nat. Chem. *8*, 958–967. https://doi.org/10.1038/nchem.2554.

S9. Gage, G.J., Kipke, D.R., and Shain, W. (2012). Whole animal perfusion fixation for rodents. J. Vis. Exp. https://doi.org/10.3791/3564.

S10. Ertürk, A., Becker, K., Jährling, N., Mauch, C.P., Hojer, C.D., Egen, J.G., Hellal, F., Bradke, F., Sheng, M., and Dodt, H.-U. (2012). Three-dimensional imaging of solvent-cleared organs using 3DISCO. Nat. Protoc. *7*, 1983–1995. https://doi.org/10.1038/nprot.2012.119.

S11. Diel, E.E., Lichtman, J.W., and Richardson, D.S. (2020). Tutorial: avoiding and correcting sample-induced spherical aberration artifacts in 3D fluorescence microscopy. Nat. Protoc. *15*, 2773–2784. https://doi.org/10.1038/s41596-020-0360-2.
